# Nanozymes-Armed Probiotic *Lactobacillus plantarum* Coordinates Ferroptosis-like Bacterial Death for Diabetic Wound Therapy

**DOI:** 10.34133/research.1258

**Published:** 2026-05-21

**Authors:** Jinjie Hou, Ruijie Fu, Mengliu Zhao, Anlai Zou, Yidan Wang, Zexiang Wang, Yunlei Xianyu

**Affiliations:** ^1^ Department of Clinical Laboratory of Sir Run Run Shaw Hospital, College of Biosystems Engineering and Food Science, Zhejiang University School of Medicine, Hangzhou 310058, People’s Republic of China.; ^2^ Key Laboratory of Precision Medicine in Diagnosis and Monitoring Research of Zhejiang Province, Sir Run Run Shaw Hospital, Hangzhou 310016, People’s Republic of China.

## Abstract

High-glucose microenvironment in diabetic wound exacerbates the risk of bacterial infections and perpetuates chronic inflammation, thereby complicating wound healing. To meet these challenges, we develop antibacterial agents by integrating probiotics with nanozymes targeting high glucose levels and multidrug-resistant bacterial infections in diabetic wound healing. We engineer probiotic *Lactobacillus plantarum* (LP) with Fe-based nanozymes containing glucose oxidase (GOx) as antibacterial agents (LP@FeG) for diabetic wound therapy. The bioactivity of LP ferments glucose in the wounds, thereby reducing local pH through lactic acid production. Besides, GOx catalyzes the conversion of glucose into gluconic acid and hydrogen peroxide, further lowering local pH. In addition, GOx stimulates the peroxidase-like activity of Fe-based nanozymes, thereby generating reactive oxygen species that attack the cellular membrane, facilitating the entry of Fe^2+^/Fe^3+^ into bacteria and inducing bacterial death through ferroptosis and lipid peroxidation. In vivo studies demonstrate that LP@FeG exhibits prolonged sterilization of methicillin-resistant *Staphylococcus aureus* with an almost 100% antibacterial rate, marked anti-inflammatory, and proangiogenic effects in diabetic wounds. This strategy resolves both the high glucose level and bacterial infections in wound healing, being promising for the treatment of diabetic wounds.

## Introduction

Diabetes, characterized by chronic hyperglycemia, has emerged as a major global public health concern, affecting approximately 425 million individuals worldwide [1–5]. The World Health Organization reports that the global diabetic population will rise to 629 million by 2045 [6–9]. As one of the most common chronic complications of diabetes, diabetic wounds pose substantial healing challenges due to elevated glucose levels, which increase the risk of bacterial infections and prolong healing processes [10–13]. Antibiotics are extensively utilized to treat bacterial infections in diabetic wounds. However, the overuse of antibiotics leads to the emergence of multidrug-resistant (MDR) bacterial strains, undermining the efficacy of conventional antibiotic therapies [14–19]. Elevated glucose levels and infections caused by MDR bacteria are the main issues in treating diabetic wounds. In response, researchers are exploring antibacterial agents and strategies to address these challenges in diabetic wounds [20,21].

The primary challenge in healing diabetic wounds is the depletion of elevated glucose levels to expedite the healing process. Probiotics, a promising category of live microorganisms, offer potential health benefits when administered in appropriate amounts [22–24]. Certain types of probiotics can regulate glucose levels, thereby minimizing the damage inflicted by hyperglycemia on wound healing [25–27]. Probiotic therapy has emerged as an effective strategy for treating inflammatory diseases by secreting diverse metabolites and antimicrobial agents [28–30]. Probiotics can modulate the wound environment, inhibit pathogens, reduce inflammation, and promote wound healing [31–34]. As a important type of probiotics, *Lactobacillus plantarum* (LP) can ferment glucose through the glycolytic pathway. LP initially converts glucose into pyruvate, which is further metabolized into lactic acid by lactate dehydrogenase (LDH). LDH catalyzes the reduction of pyruvate to lactate and oxidizes nicotinamide adenine dinucleotide to reduced nicotinamide adenine dinucleotide, thus effectively lowering the environmental pH through acidification. Besides LDH, glucose oxidase (GOx) can also convert glucose into gluconic acid and hydrogen peroxide (H_2_O_2_), reducing glucose levels and providing antibacterial benefits for diabetic wound healing.

Besides elevated glucose levels, the healing process of diabetic wounds is hampered by MDR bacterial infections. To address this issue, nanozyme-based therapy has shown great potential for treating bacterial infections [35,36]. Unlike conventional antibiotics, nanozymes function through multiple mechanisms without inducing bacterial resistance, owing to their small sizes and exceptional catalytic properties [37–40]. Nanozymes can generate reactive oxygen species (ROS) that directly damage bacterial cell membranes, proteins, and nucleic acids. In addition, they alleviate inflammation and oxidative stress that accelerates the wound healing process. Nanozymes with antibacterial capabilities are categorized into metal-based, carbon-based, metal-organic-framework-based, and single-atom-based nanomaterials. Among them, Fe-based nanozymes (Fe NZs) possess robust catalytic properties that mimic natural enzymes such as peroxidases (PODs) and oxidases. Iron plays a critical role in bacterial survival and metabolism, affecting essential processes such as DNA synthesis, energy metabolism, and redox reactions [41,42]. The dual role of iron, serving as both an enzymatic cofactor and a metabolic target, makes Fe NZs promising for antibacterial applications. They can induce ferroptosis, a form of programmed cell death characterized by Fe-dependent lipid peroxidation, resulting in membrane damage and bacterial death. The synergistic effect of nanozymes and ferroptosis provides a promising strategy for combating bacterial infections, given the enhanced uptake of iron ions in bacteria that facilitates lipid peroxidation [43,44].

**Fig. 1.**
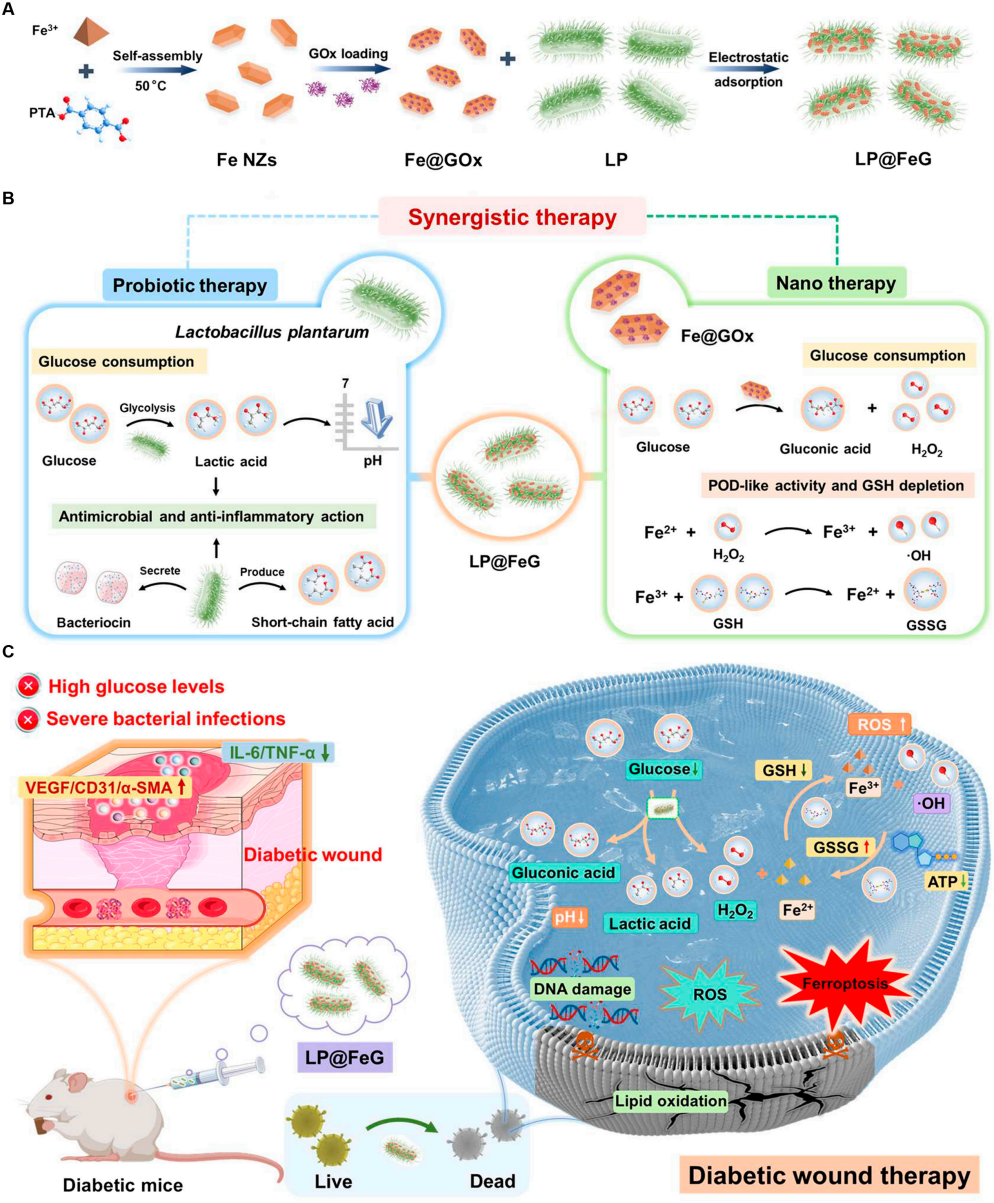
Schematic illustration of the design of *L. plantarum* and Fe-based nanozymes loaded with glucose oxidase (LP@FeG) and the synergistic therapeutic mechanism for diabetic wounds. (A) Synthesis of the LP@FeG composite. PTA, *p*-phthalic acid. (B) Synergistic therapeutic mechanisms of LP@FeG. (C) Application of LP@FeG in diabetic wound therapy in mice.

Herein, we developed a composite of probiotic LP and Fe NZs loaded with GOx (LP@FeG) for the synergistic therapy and healing of diabetic wounds (Fig. 1). First, probiotic LP modulated elevated glucose levels in diabetic wounds by converting glucose into lactic acid via glycolysis. Second, GOx within LP@FeG effectively depleted glucose levels in diabetic wounds, yielding gluconic acid and H_2_O_2_. Third, Fe NZs catalyzed the conversion of H_2_O_2_ into ROS and induced ferroptosis in bacteria, thereby accelerating lipid peroxidation and disrupting membrane integrity. Consequently, LP@FeG modulated the microenvironment in diabetic wounds and eradicated MDR bacteria, thereby accelerating wound healing in diabetic mice. In addition, probiotic LP alleviated inflammation in diabetic wound, further improving the overall wound conditions and expediting the healing process. This therapeutic strategy boosts the therapeutic efficacy by leveraging multiple mechanisms, effectively addressing the issues in diabetic wounds characterized by high-glucose environments and bacterial infections.

## Results and Discussion

### Preparation and characterization of LP@FeG

**Fig. 2.**
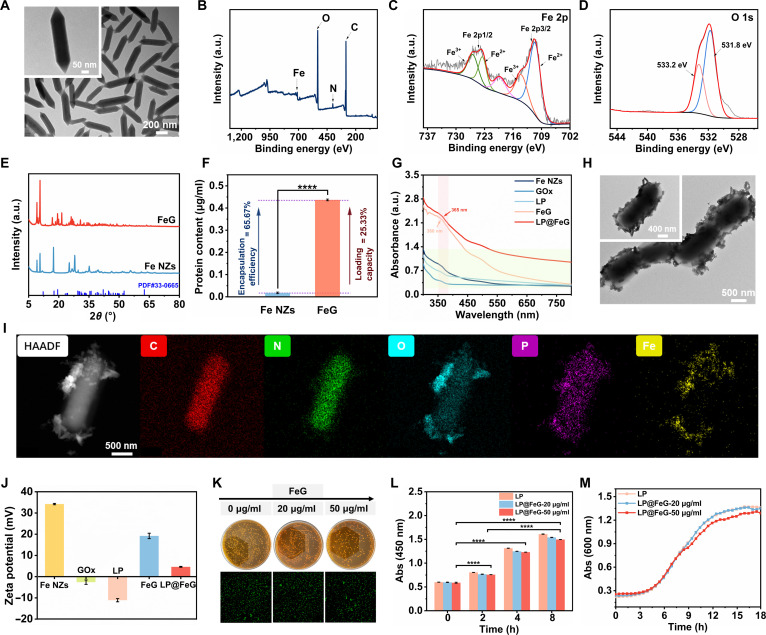
Characterizations of *L. plantarum* and Fe-based nanozymes (Fe NZs) loaded with glucose oxidase (GOx) (LP@FeG). (A) Transmission electron microscopy (TEM) and magnified images of Fe NZs. (B) X-ray photoelectron spectroscopy (XPS) spectrum of Fe NZs. XPS spectra for (C) Fe 2p and (D) O 1s in Fe NZs. (E) X-ray diffraction (XRD) patterns of Fe NZs and FeG. (F) Encapsulation efficiency and loading capacity of GOx on FeG. (G) UV–vis spectra of Fe NZs, GOx, LP, FeG, and LP@FeG. (H) TEM and magnified images of LP@FeG. (I) High-angle annular dark-field (HAADF)-scanning TEM images and corresponding elemental maps of C, N, O, P, and Fe distribution of LP@FeG. (J) Zeta potentials of Fe NZs, GOx, LP, FeG, and LP@FeG. (K) Spread-plate images of LP cultured with FeG for 12 h. (L) Cell viability monitored at 2-h intervals. Abs, absorbance. (M) Growth curves of LP cultured with FeG over 18 h.

We synthesized Fe NZs-armed probiotic LP using a one-step assembly approach. First, Fe NZs were prepared via a straightforward self-assembly method. Transmission electron microscopy (TEM) characterization of Fe NZs revealed a shuttle-like nanostructure (Fig. 2A). X-ray photoelectron spectroscopy (XPS) analysis showed that Fe NZs primarily contained Fe, C, N, and O (Fig. 2B). Analysis of Fe and O oxidation states confirmed the coexistence of Fe^2+^ and Fe^3+^ with distinctive peaks at 710.68/723.58 and 714.18/725.98 eV for Fe 2p3/2 and Fe 2p1/2 orbitals, respectively (Fig. 2C). The Fe^2+^/Fe^3+^ ratio was calculated to be 1.44:1 based on the peak area. In addition, peaks at 531.78 and 533.28 eV corresponded to O 1s orbitals (Fig. 2D). X-ray diffraction (XRD) analysis of the crystal structure of Fe NZs and FeG showed that GOx loading had no impact on the structural characteristics, consistent with TEM images (Fig. 2E and Fig. S1). The loading capacity and encapsulation efficiency of GOx within FeG were quantified as 25.33% and 65.67%, respectively (Fig. 2F). The composite of LP@FeG was achieved through electrostatic adsorption, as confirmed by ultraviolet–visible (UV–vis) absorption spectra and TEM images (Fig. 2G and H). The UV–vis spectra indicated the conjugation of FeG onto LP, as evidenced by the distinct absorption peak at approximately 365 nm. The LP@FeG composite surpassed the absorbance of individual LP and FeG, along with broad absorption features from 450 to 800 nm as a result of electronic transitions after conjugation. Energy-dispersive x-ray spectroscopy analysis of the high-angle annular dark-field (HAADF) image confirmed the uniform distribution of C, N, O, P, and Fe elements on the probiotic LP (Fig. 2I and Fig. S2). The zeta potential of Fe NZs decreased from 34.2 to 19.2 mV after loading with negatively charged GOx. Furthermore, the zeta potential of FeG shifted from 19.2 to 4.6 mV after electrostatic adsorption onto probiotic LP (Fig. 2J).

To evaluate the effect of FeG modification on probiotic LP activity, we conducted coculture at 37 °C using different amounts of FeG (0, 20, and 50 μg/ml) for 0, 2, 4, and 8 h. The viability of LP in LP@FeG was determined by plate counting, live/dead staining, and Cell Counting Kit-8 (CCK-8) assays. The plates and fluorescence microscopy images showed a slight reduction in viability with elevated FeG concentration although LP remained largely viable (Fig. 2K). The CCK-8 assay results demonstrated a modest reduction in bacterial metabolic activity with elevated FeG concentration (Fig. 2L). The growth curve showed that LP@FeG experienced a slight delay in reaching the exponential phase at 50 μg/ml, although the overall growth remained robust. FeG exerted a negligible influence on the growth curves of LP after an 18-h incubation (Fig. 2M). To identify the ideal type of probiotics for therapy, we selected six different strains and prepared the probiotics@FeG library, including LP@FeG, *Escherichia coli* Nissle 1917@FeG (EcN@FeG), *Bacillus subtilis*@FeG (B.sub@FeG), *Bacillus cereus*@FeG (B.cer@FeG), *Bacillus licheniformis*@FeG (B.lic@FeG), and *Saccharomyces cerevisiae*@FeG (S.cer@FeG) (Fig. S3).

### Cascade catalytic properties of LP@FeG

**Fig. 3.**
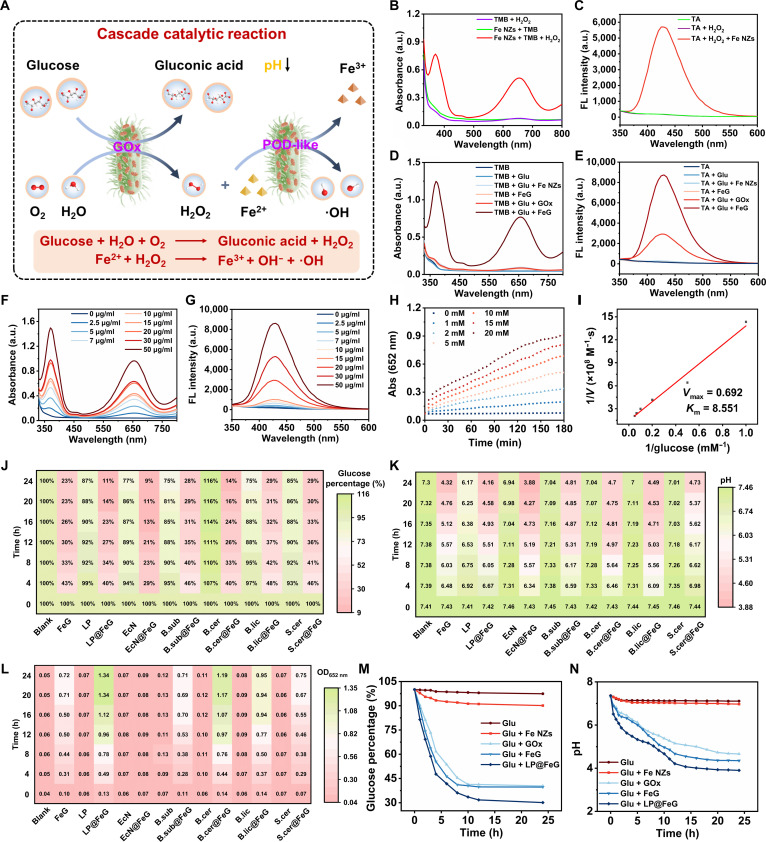
Study on the cascade catalytic reactions of *L. plantarum* and Fe-based nanozymes (Fe NZs) loaded with glucose oxidase (LP@FeG). (A) Schematic illustration of the cascade catalytic reactions. (B) Ultraviolet–visible (UV–vis) absorption spectra and (C) fluorescence (FL) spectra of Fe NZs under different conditions. (D) UV–vis absorption spectra and (E) fluorescence spectra of FeG under different conditions. (F) UV–vis absorption spectra and (G) fluorescence spectra of FeG at different concentrations. (H) Time-dependent absorbance changes catalyzed by FeG and glucose at different concentrations. (I) Lineweaver–Burk fitting of Michaelis–Menten curve of ·OH generation velocities against glucose concentrations. Heatmaps indicated the variation in (J) glucose concentration, (K) pH, and (L) optical density at 652 nm (OD_652 nm_) for probiotics and probiotics@FeG. Variation in (M) glucose and (N) pH under different conditions.

We studied the mechanism of the cascade catalytic reaction in LP@FeG (Fig. 3A). Briefly, GOx catalyzed the conversion of glucose into H_2_O_2_, and the intermediate H_2_O_2_ reacted with Fe^2+^ to generate hydroxyl radicals (·OH). To evaluate the POD-like activity of Fe NZs, we used 3,3′,5,5′-tetramethylbenzidine (TMB) as a colorimetric substrate. The oxidation of TMB by H_2_O_2_ yielded a product with a characteristic absorbance at 652 nm, confirming the excellent POD-like activity of Fe NZs (Fig. 3B). Notably, the presence of H_2_O_2_ in an acidic environment enhanced the POD-like activity of Fe NZs, facilitating the generation of ·OH. In the presence of H_2_O_2_ and Fe NZs, nonfluorescent terephthalic acid (TA) was converted to highly fluorescent 2-hydroxyterephthalic acid (TAOH) with an emission peak at 425 nm, confirming the generation of ·OH (Fig. 3C). The optimal conditions for the POD-like activity of Fe NZs were identified at pH 4 and 37 °C (Fig. S4). We investigated the steady-state kinetics of TMB/H_2_O_2_ as substrates (Fig. S5). The Michaelis constant (*K*_m_) values were 1.426 × 10^−3^ and 2.944 × 10^−3^ M, while the maximum velocity (*V*_max_) values were 10.412 × 10^−7^ and 12.797 × 10^−7^ M/s, respectively. Fe NZs exhibited superior POD-like activity in comparison to other nanomaterials reported under similar conditions (Table S1).

We investigated the FeG-catalyzed cascade reaction. GOx catalyzed the oxidation of glucose into gluconic acid, which decreased the pH value and enhanced the POD-like activity of Fe NZs. The absorbance at 652 nm increased in the presence of glucose and FeG, indicating the GOx-POD cascade reaction (Fig. 3D). We further confirmed the cascade catalytic reaction through a fluorescent approach (Fig. 3E). FeG catalyzed the conversion of nonfluorescent TA into highly fluorescent TAOH in the presence of glucose, indicating the generation of ·OH. In addition, we evaluated the impact of FeG concentration on the catalytic activity, showing that the absorbance at 652 nm increased with the elevated concentration of FeG (Fig. 3F). Meanwhile, fluorescence assays confirmed that ·OH production correlated with the concentration of FeG (Fig. 3G). We quantitatively analyzed the steady-state kinetics of the GOx-like activity of FeG by adding glucose (Fig. S6). Increasing glucose concentration and reaction time led to an increased absorbance at 652 nm (Fig. 3H). The *V*_max_ value of glucose was 6.92 × 10^−8^ M/s, and the *K*_m_ value was 8.551 mM (Fig. 3I). To continuously monitor the pH change, we used methyl red as a pH indicator that remained yellow in the control groups (Figs. S7 and S8). In contrast, the incubation of FeG with glucose resulted in a distinct color change from yellow to red, confirming that FeG effectively lowered the pH value.

Owing to the metabolic capabilities of probiotic LP in glucose utilization and lactic acid production, we developed the LP@FeG composite by incorporating FeG into LP. Meanwhile, we prepared other probiotics@FeG composites for comparison. We studied the impact of LP loading on glucose consumption and pH regulation. LP metabolized glucose via the glycolytic pathway, initially converting it into pyruvate, which was further processed by LDH into lactic acid, leading to local glucose depletion and pH reduction. Simultaneously, GOx in LP@FeG catalyzed glucose oxidation, producing gluconic acid and H_2_O_2_, further reducing glucose concentration and enhancing acidity. Glucose levels in LP@FeG decreased by approximately 11% over time, lower than those in FeG and other probiotics@FeG (Fig. 3J and M). The pH value within LP@FeG decreased to 4.16, close to the optimal pH for POD-like activity (Fig. 3K and N). The LP alone group exhibited a moderate pH decrease due to lactic acid production but failed to sustain marked acidity over time. The Fe NZs alone and phosphate-buffered saline (PBS) groups displayed negligible changes, confirming that Fe NZs without GOx or probiotics could not acidify the environment. The FeG alone group showed an initial pH decrease through gluconic acid production but exhibited pH rebound due to glucose depletion and lack of continuous acid generation. In contrast, LP@FeG maintained a lower and more stable pH (~4.16) throughout the observation period, demonstrating the synergistic and sustained acidification achieved by combining probiotic fermentation and enzymatic catalysis. The sustained presence of glucose and the resulting acidic microenvironment maintained the enzymatic activity of Fe NZs, ensuring continuous ROS production. Lactic acid from probiotic metabolism stabilized the acidic environment, enhancing catalytic efficiency of Fe NZs. In the presence of glucose, LP@FeG exhibited optimal POD-like activity based on absorbance at 652 nm, surpassing that of FeG and other probiotics@FeG (Fig. 3L). To determine the optimal concentrations of glucose and LP@FeG for maximal catalytic efficiency, we investigated their effects on the regulation of glucose level and pH value (Figs. S9 and S10). LP@FeG at a concentration of 50 μg/ml resulted in a pH value less than 4.5. These results underscored the potential of LP@FeG in cascade catalysis, particularly in enhancing the POD-like activity and ·OH generation. The glucose consumption with LP@FeG (50 μg/ml) was more than 80%, demonstrating sustained metabolic activity (Fig. S11A). The pH value decreased to below 4.5 due to acid production, creating an environment that enhanced the antibacterial activity (Fig. S11B). The absorbance at 652 nm showed a minimal decline, indicating the retention of POD-like activity (Fig. S11C). In addition, we assessed the stability of LP@FeG over 8 d (Fig. S12). The colony-forming unit (CFU) count for LP gradually decreased by approximately 24% from 2.5 × 10^6^ (day 0) to 1.9 × 10^6^ (day 8). The glucose percentage increased from 12.0% (day 0) to 16.1% (day 30), reflecting a decrease in metabolic activity. The pH value increased from 4.2 to 4.8, indicating a slight reduction in acid production. There was a decline in the POD-like activity, with absorbance at 652 nm decreasing from 1.35 to 1.29 during the storage. These results indicated that while storage affected the viability of LP, glucose metabolism, and enzymatic activity, the effects remained moderate. LP@FeG retained considerable bacterial viability and functional activity, underscoring its stability during storage for potential practical applications.

### Antibacterial performance of LP@FeG in vitro

**Fig. 4.**
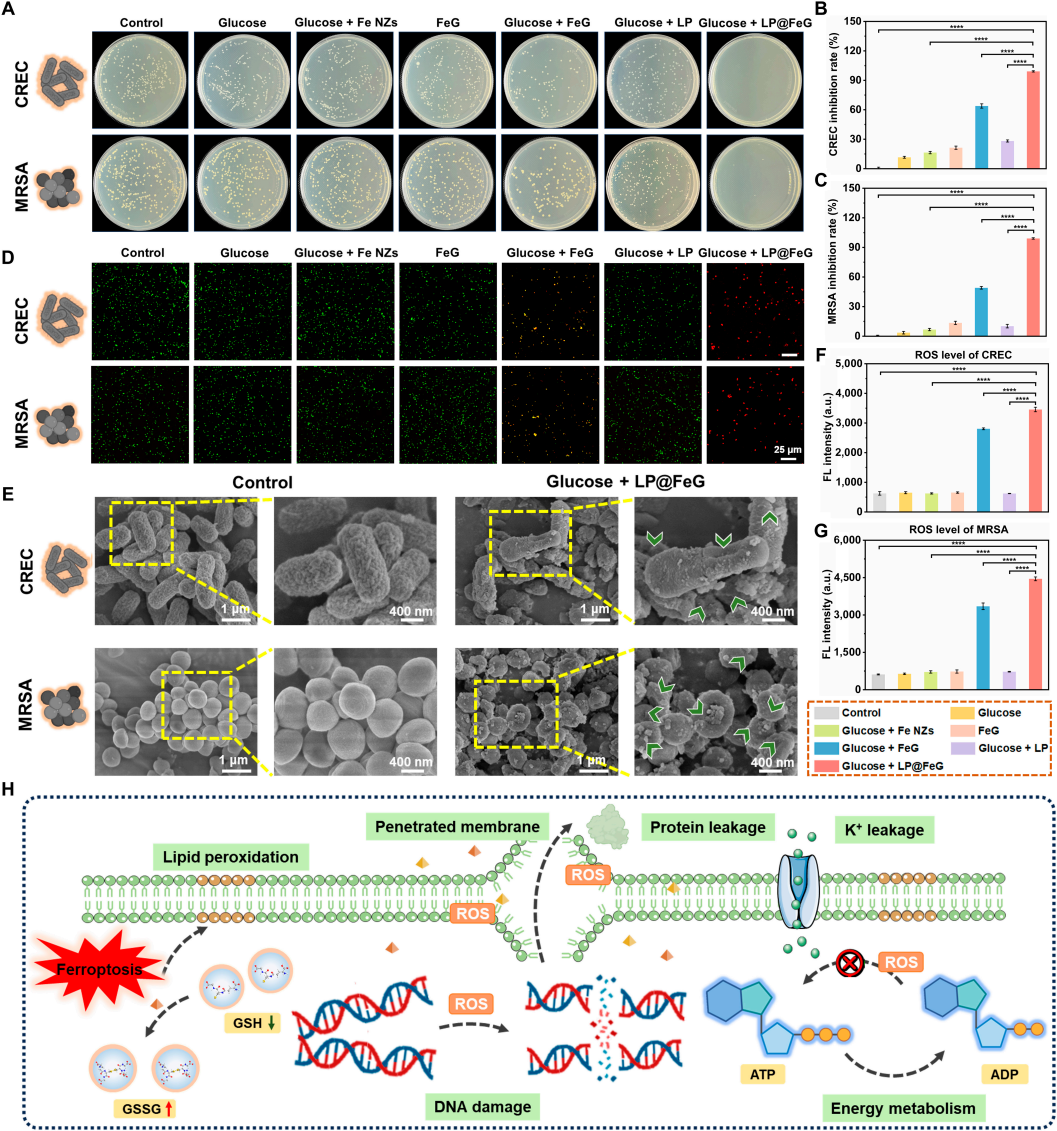
The antibacterial activities of *L. plantarum* and Fe-based nanozymes loaded with glucose oxidase (LP@FeG) in vitro. (A) Digital images of carbapenem-resistant *E. coli* (CREC) and methicillin-resistant *S. aureus* (MRSA) colonies with different treatments. Antibacterial effects of LP@FeG against (B) CREC and (C) MRSA. (D) Live/dead staining of CREC and MRSA with different treatments. (E) Scanning electron microscopy (SEM) images of untreated or glucose and LP@FeG-treated CREC and MRSA. Green arrows indicated damaged cells. Fluorescence quantification of reactive oxygen species (ROS) in (F) CREC and (G) MRSA. (H) Schematic illustration of the antibacterial mechanism of LP@FeG. ADP, adenosine diphosphate. Data are presented as means ± SD. *n* = 3. ****P < 0.0001..

MDR bacterial infections pose serious risks to diabetic wounds, and the effective elimination is crucial for wound healing. LP@FeG facilitated the production of ROS, thereby enhancing its antibacterial efficacy. We assessed the antibacterial efficacy of LP@FeG against MDR bacterial strains including carbapenem-resistant *E. coli* (CREC) and methicillin-resistant *Staphylococcus aureus* (MRSA). After treatment with FeG and LP@FeG, the colony numbers for CREC and MRSA were reduced (Fig. 4A). Quantitative analysis revealed that the bacterial inhibition rates were 99.13% and 99.08% for LP@FeG against CREC and MRSA, respectively (Fig. 4B and C).

We investigated the antibacterial performance of LP@FeG and other probiotics@FeG, showing that LP@FeG exhibited the highest antibacterial efficiency against CREC and MRSA (Fig. S13). LP@FeG at a concentration of 50 μg/ml effectively eliminated approximately 100% of CREC and MRSA (Fig. S14). Higher concentrations (>50 μg/ml) of LP@FeG showed no additional benefits since increasing the concentration could not further enhance the antibacterial efficacy (Fig. S15). The minimal inhibitory concentration of LP@FeG was set as 50 μg/ml. Live/dead fluorescence staining showed red fluorescence in the LP@FeG group that differed from green or mixed green/red fluorescence in other groups, further confirming the prominent antibacterial efficacy (Fig. 4D). Scanning electron microscopy (SEM) images revealed the antibacterial properties of LP@FeG through alterations of bacterial morphology. Native CREC and MRSA exhibited rod and spherical morphologies with smooth surfaces (Fig. 4E). In the presence of glucose, LP@FeG induced localized contraction and distortion of the membrane (green arrow). We used 2′,7′-dichlorodihydrofluorescein diacetate (DCFH-DA) staining to monitor ROS generation in CREC and MRSA. ROS levels increased when CREC and MRSA were treated with glucose and FeG/LP@FeG, while other groups showed negligible ROS signals (Fig. 4F and G). This suggested that FeG and LP@FeG initiated the cascade catalytic reaction in the presence of glucose, enhancing ROS accumulation within bacterial cells. We further investigated the antibacterial mechanism of LP@FeG against CREC and MRSA by assessing multiple parameters, including lipid peroxidation, protein leakage, K^+^ leakage, nucleic acid leakage, DNA damage, and adenosine triphosphate (ATP) level (Fig. 4H). Quantitative analyses provided further validation for the aforementioned results (Figs. S16 and S17). Therefore, LP@FeG exhibited potent antibacterial activity through the generation of ROS, destruction of bacterial structures, and leakage of intracellular contents. Moreover, we evaluated the efficacy of LP@FeG against other common wound pathogens, encompassing *Pseudomonas aeruginosa*, *E. coli*, and *S. aureus*. LP@FeG exhibited substantial antibacterial activity against these strains, highlighting its potential as a broad-spectrum antibacterial agent (Fig. S18).

### Antibacterial property of LP@FeG via ferroptosis

**Fig. 5.**
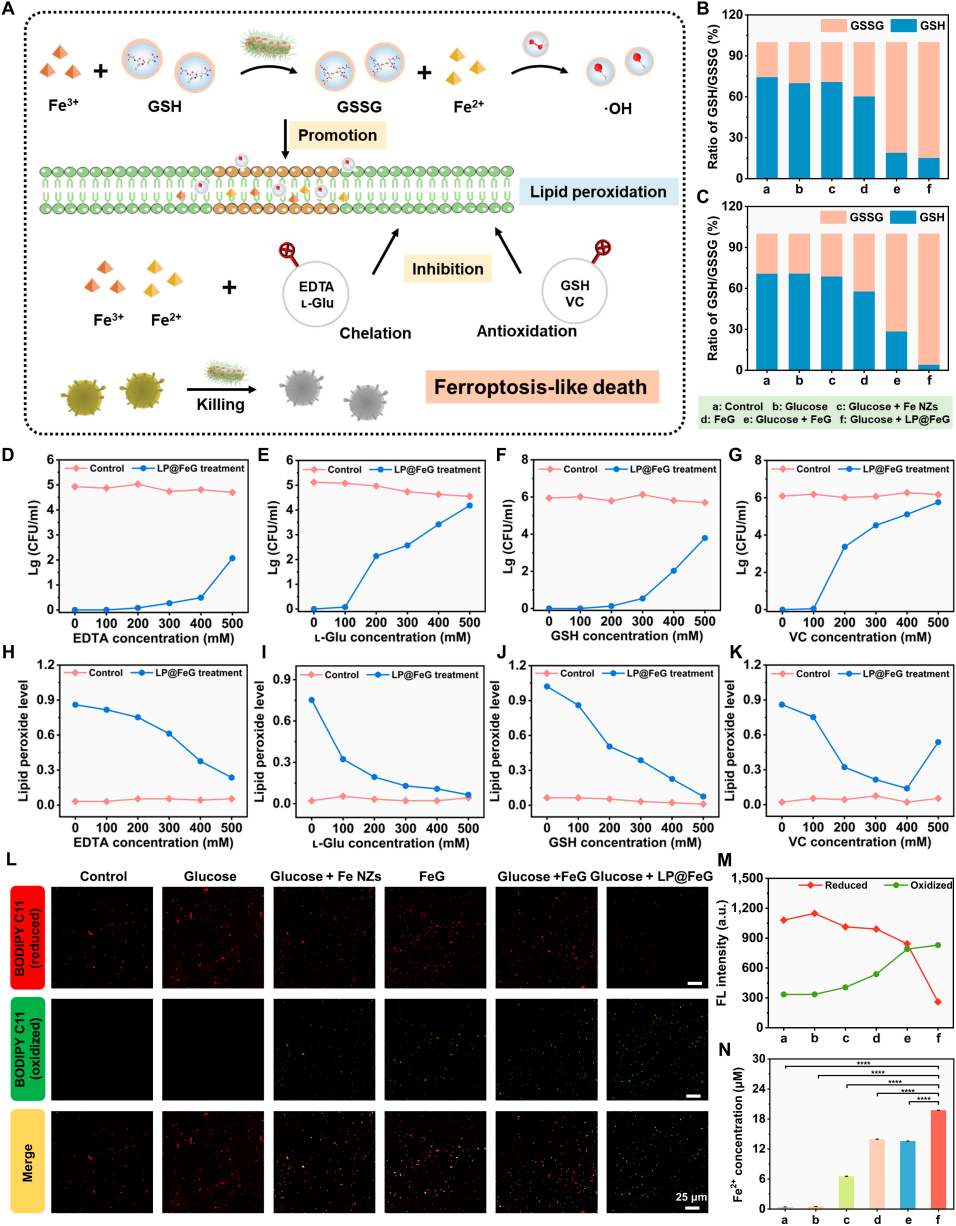
Antibacterial properties of *L. plantarum* and Fe-based nanozymes loaded with glucose oxidase (LP@FeG) mediated by ferroptosis. (A) Schematic illustration of the mechanism of ferroptosis-like death. Ratio of reduced glutathione (GSH)/oxidized glutathione (GSSG) in (B) carbapenem-resistant *E. coli* (CREC) and (C) methicillin-resistant *S. aureus* (MRSA) after different treatments. Inhibition effects of (D) ethylenediaminetetraacetic acid (EDTA), (E) l-glutamic acid (l-Glu), (F) GSH, and (G) vitamin C (VC) on MRSA colonies treated by LP@FeG. Lg, log_10_. (H to K) Lipid peroxidation of MRSA with different treatments. (L) Monitoring of lipid peroxidation in MRSA using BODIPY 581/591 C11. (M) Fluorescence quantification of lipid peroxidation in MRSA. (N) Fe^2+^ concentration in MRSA with different treatments. Data are presented as means ± SD. *n* = 3. ****P < 0.0001.

In addition to ROS, LP@FeG induced ferroptosis-like bacterial death (Fig. 5A). We detected the ratio of reduced glutathione (GSH) to oxidized glutathione (GSSG) in both CREC and MRSA. Under acidic conditions, Fe^3+^ oxidizes GSH to GSSG, disrupting the balance between GSSG and GSH. The GSH/GSSG ratio for CREC and MRSA decreased from 0.74 and 0.71 to 0.15 and 0.04, respectively (Fig. 5B and C). Ethylenediaminetetraacetic acid (EDTA), a ferroptosis inhibitor, was added to validate ferroptosis-like death of bacteria. EDTA as an iron chelator reduced the bacterial killing and lipid peroxidation in MRSA (Fig. 5D and H) and CREC (Fig. S19). Similarly, other iron-binding agents such as l-glutamic acid (l-Glu) (Fig. 5E and I), antioxidants such as GSH (Fig. 5F and J) and vitamin C (VC) (Fig. 5G and H) inhibited the antibacterial activity and lipid peroxidation induced by LP@FeG. In addition, we used BODIPY C11 to monitor the lipid peroxidation in MRSA (Fig. 5L) and CREC (Fig. S20). BODIPY C11 emitted a red fluorescence, while oxidation of the polyunsaturated butadiene group shifted the emission peak with a green fluorescence. Quantitative results indicated enhanced green fluorescence and elevated levels of bacterial lipid peroxidation after treatment with glucose and LP@FeG (Fig. 5M and Fig. S21). Furthermore, both CREC and MRSA treated with LP@FeG accumulated high levels of Fe^2+^ (20.19 and 19.76 μM), characteristic of ferroptosis-like death (Fig. 5N and Fig. S22).

### Antibacterial mechanism of LP@FeG by transcriptomic analysis

**Fig. 6.**
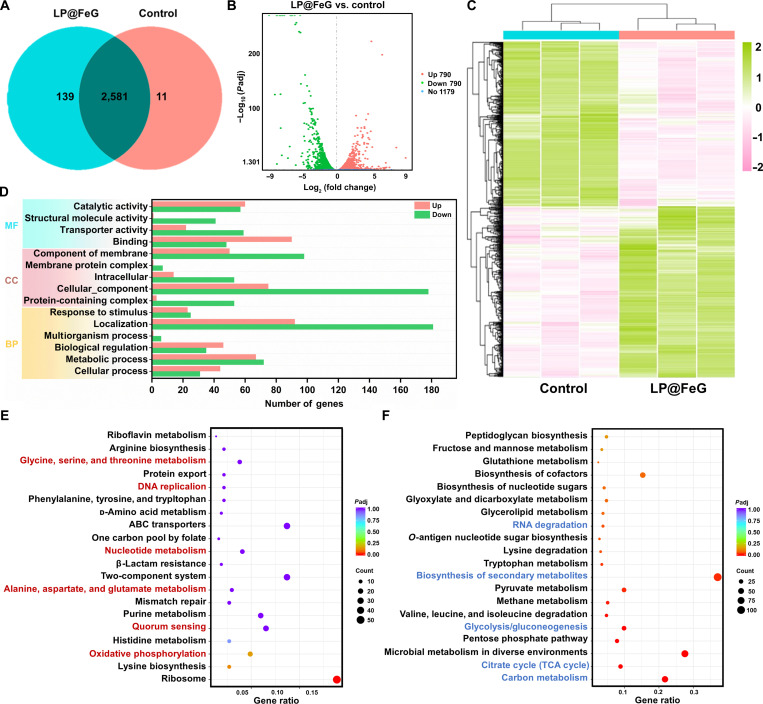
Antibacterial mechanisms of *L. plantarum* and Fe-based nanozymes loaded with glucose oxidase (LP@FeG) through transcriptomic analysis. (A) Venn diagram of differentially expressed genes (DEGs) between the control and LP@FeG groups. (B) Volcano plot of total DEGs in methicillin-resistant *S. aureus* (MRSA) in control and the LP@FeG group. (C) Heatmap of DEGs between control and LP@FeG groups. (D) Gene Ontology enrichment analysis of DEGs between control and LP@FeG groups. BP, biological process; CC, cellular component; MF, molecular function. (E) Up-regulated and (F) down-regulated DEGs enriched in the Kyoto Encyclopedia of Genes and Genomes pathway. ABC, adenosine-triphosphate-binding cassette transporter; TCA, tricarboxylic acid.

We conducted a transcriptomic analysis to investigate gene expressions in MRSA after treatment with LP@FeG. The gene expression diagram showed high correlation within and between groups, ensuring the reliability of differential gene analysis (Fig. S23). Venn diagram analysis revealed 2,581 genes expressed in the control and LP@FeG groups, with 11 genes exclusive to the control and 139 genes exclusive to the LP@FeG group (Fig. 6A). Volcano plot analysis identified 790 up-regulated and 790 down-regulated genes in the LP@FeG group compared to the control (Fig. 6B). The clustering heatmap of differential gene expression revealed that treatment with LP@FeG altered gene expression, affecting biological processes including ribosome replication, recombination, and repair (Fig. 6C). These processes included up-regulation of *dnaA*, down-regulation of *recF*, and up-regulation of *infB*, *trmD*, *rpsL*, *rpmG*, *rplL*, *nrdF*, and *thrS* genes. In lipid transport and metabolism, *scpA* was down-regulated, while *ggt* and *MK631_RS02420* were up-regulated, suggesting the inhibition of cysteine metabolism, depletion of glutathione, and increased lipid peroxidation. The up-regulation of transferrin-associated genes such as *feoB* indicated an enrichment of cellular iron, and the up-regulation of *narH* relating to the bacterial respiratory chain indicated the ferroptosis. Meanwhile, genes related to ATP production and transformation (*MK631_RS04590*, *atpA*, *atpB*, *atpD*, *atpE*, and *atpG*) were down-regulated, suggesting marked inhibition of ATP production in MRSA after treatment with LP@FeG. The up-regulation of *MK631_RS02205* indicated that LP@FeG induced oxidative stress, subsequently inhibiting MRSA growth and replication. Gene Ontology enrichment analysis demonstrated the down-regulation across all terms including biological processes, cellular components, and molecular functions (Fig. 6D). Kyoto Encyclopedia of Genes and Genomes enrichment analysis highlighted that up-regulated genes were predominantly enriched in metabolic pathways, including glycine, serine, and threonine metabolism, nucleotide metabolism, as well as genetic processes such as DNA replication and oxidative phosphorylation (Fig. 6E). In contrast, down-regulated genes were in pathways such as RNA degradation and glycolysis (Fig. 6F). The transcriptomic analysis indicated multiple regulatory mechanisms exerted by LP@FeG on MRSA, suggesting that LP@FeG effectively induced bacterial death through mechanisms similar to ROS production and ferroptosis, ultimately resulting in MRSA death [45,46].

### Treatment efficacy of LP@FeG on diabetic wounds in vivo

**Fig. 7.**
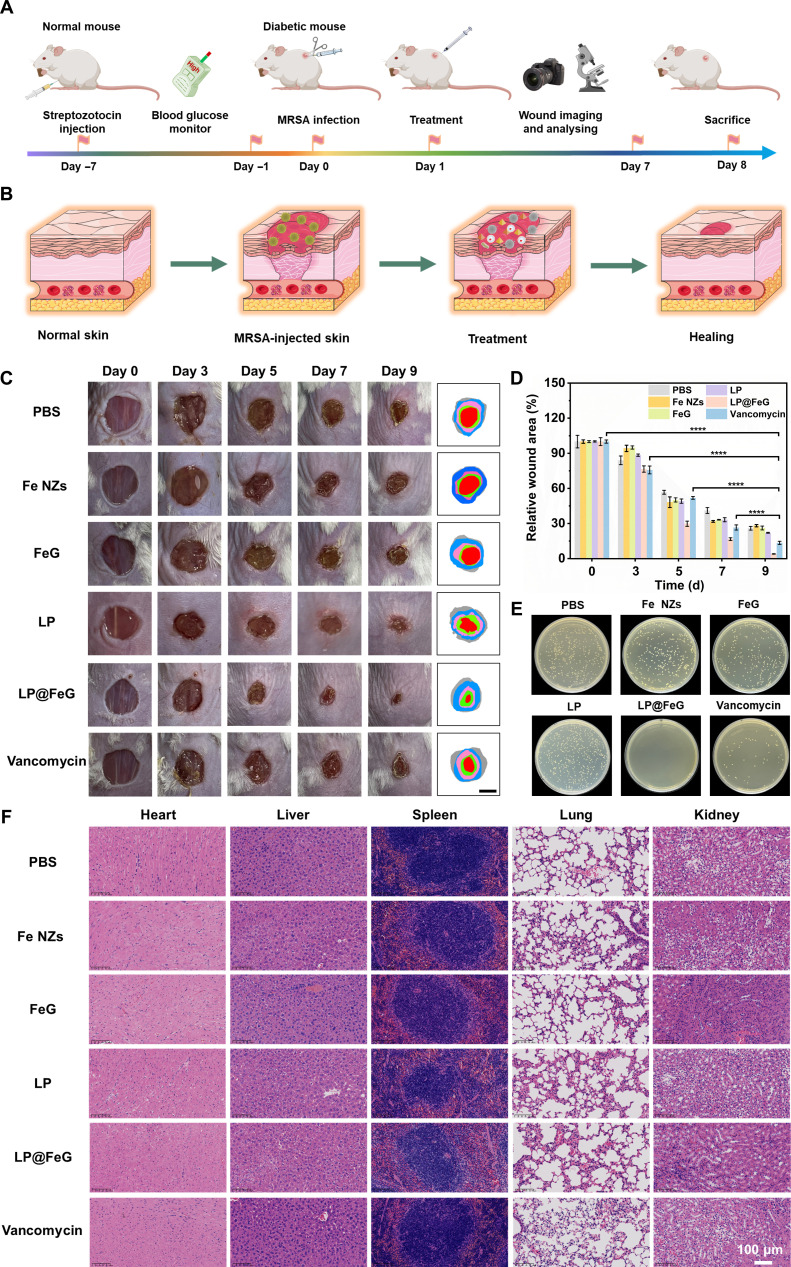
Therapeutic potential of *L. plantarum* and Fe-based nanozymes loaded with glucose oxidase (LP@FeG) in diabetic wound models. (A) Schematic diagram of the in vivo experimental procedure. (B) Schematic diagram of the healing of diabetic wound in a mouse model. (C) Sequential photographs of wound healing from various groups after 0, 3, 5, 7, and 9 d. (D) Quantitative analysis of wound areas in various groups after 0, 3, 5, 7, and 9 d. (E) Images of bacterial colonies from tissues at surgical sites. (F) Hematoxylin and eosin (H&E)-stained sections of the major organs in various groups. Data are presented as means ± SD. *n* = 6. ****P < 0.0001.

Human umbilical vein endothelial cells (HUVECs), RAW 264.7 cells, and NIH/3T3 cells are widely utilized to assess the biocompatibility of nanomaterials in wound-healing studies. During the wound healing process, fibroblasts are essential for generating and remodeling the new extracellular matrix and collagen structures, breaking down fibrin clots, and facilitating wound contraction, while macrophages play a pivotal role in modulating the inflammatory response and healing environment. To confirm the biocompatibility, we evaluated the cytotoxicity of LP@FeG on HUVECs, RAW 264.7 cells, and NIH/3T3 cells following treatment at an equivalent concentration of 200 μg/ml for 24 and 48 h. The cell viability in all the groups was higher than 95%, demonstrating the low toxicity of LP@FeG (Fig. S24). We further examined the biocompatibility of LP@FeG using fresh mouse blood. Both direct observation and quantification revealed negligible hemolysis, confirming the biocompatibility of LP@FeG (Fig. S25). To evaluate the therapeutic potential of LP@FeG in vivo, we established a diabetic wound model in mice. The blood glucose levels were over 16.7 mmol/l via intraperitoneal injections of streptozotocin for 5 consecutive days (Fig. S26). Subsequently, we created an MRSA-infected wound on the back of mice using a standardized round punch (Fig. 7A and B). The diabetic mice were randomly divided into 6 treatment groups: (a) control, (b) Fe NZs, (c) FeG, (d) LP, (e) LP@FeG, and (f) vancomycin. Throughout the healing process, we monitored the wound area of each treatment group every 2 d. The infected wound in the LP@FeG group had the smallest area on day 9, indicating the most rapid healing (Fig. 7C). Quantitative results demonstrated that the wound closure rate of the LP@FeG group was 96.03%. In contrast, the wound closure rates of the control, Fe NZ, FeG, LP, and vancomycin groups were 74.09%, 71.87%, 73.86%, 77.97%, and 86.57%, respectively, suggesting the superior efficacy of LP@FeG in wound healing (Fig. 7D). To further evaluate the antibacterial efficacy in MRSA-infected diabetic wounds, we determined the number of bacteria using the spread plate method (Fig. 7E). By the ninth day postsurgery, the antibacterial rate against MRSA in wounds reached 100% in the LP@FeG group, confirming its potent antibacterial effects (Fig. S27). These results demonstrated the therapeutic potential of LP@FeG in accelerating the repair of diabetic wounds. We conducted histopathological analysis of major organs to assess the potential toxicity. After 9 d, no marked histopathological lesions were observed (Fig. 7F). Besides, the weight of mice after treatment had no marked changes, ensuring the safety of the therapeutic strategy (Fig. S28).

**Fig. 8.**
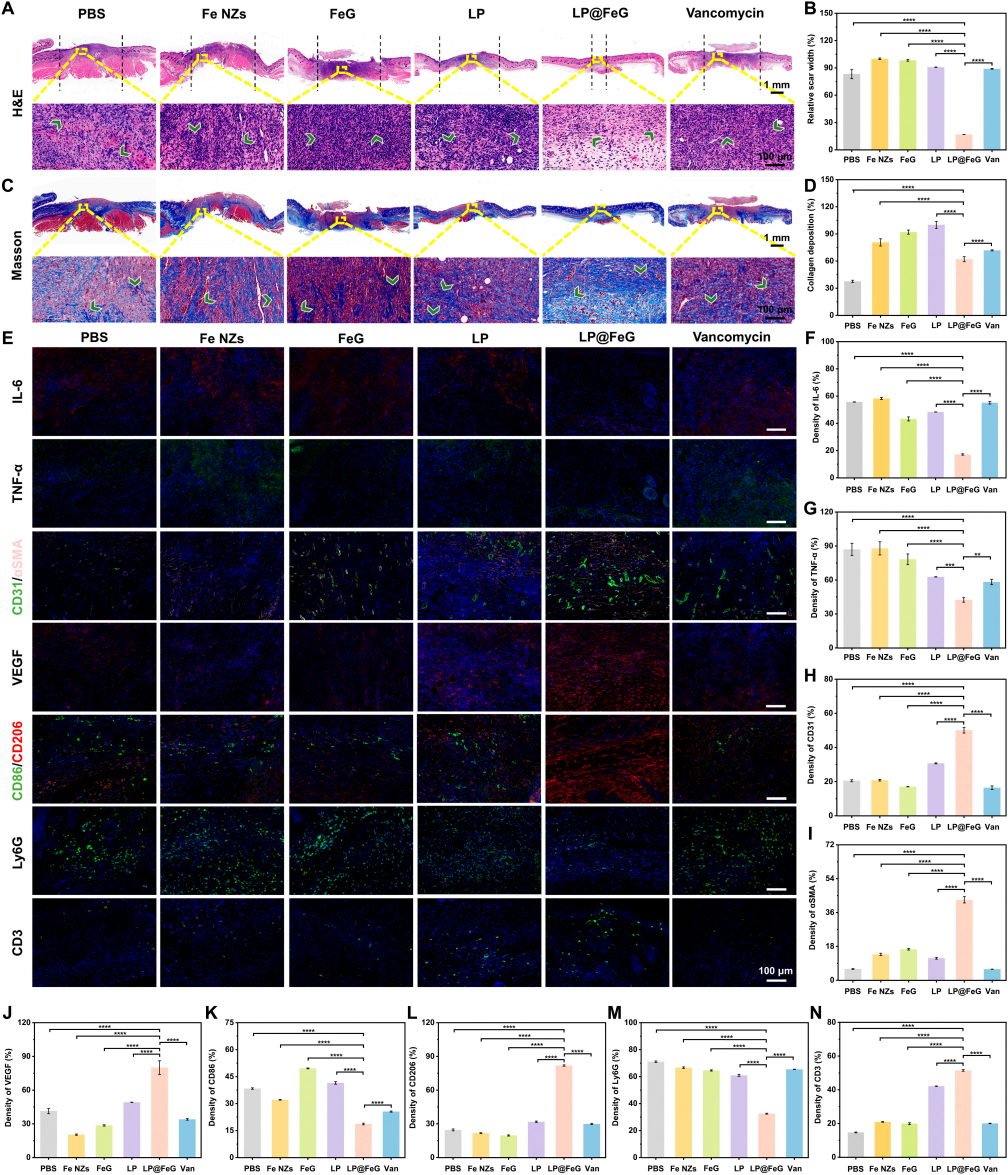
Histological studies of the skin tissues in diabetic wound models. (A) Hematoxylin and eosin (H&E) staining images of wound tissues in different groups on day 9. (B) The scar width of H&E staining images in different groups. (C) Masson staining images of wound tissues in different groups on day 9. (D) Quantitative analysis of percentage collagen deposition of the Masson staining. (E) Immunohistochemical staining for interleukin-6 (IL-6), tumor necrosis factor-α (TNF-α), cluster of differentiation 31 (CD31)/α-smooth muscle actin (αSMA), vascular endothelial growth factor (VEGF), CD86/CD206, lymphocyte antigen 6 complex, locus G (Ly6G), and CD3 on day 9. Statistical analysis of (F) IL-6, (G) TNF-α, (H) CD31, (I) αSMA, (J) VEGF, (K) CD86, (L) CD206, (M) Ly6G, and (N) CD3 on day 9. Data are presented as means ± SD. *n* = 6. **P < 0.01; ***P < 0.001; ****P < 0.0001.

We used hematoxylin and eosin (H&E) and Masson staining to elucidate the inflammatory response, collagen deposition, and angiogenesis efficiency during diabetic wound regeneration. We performed a comprehensive analysis of the wound site on day 7, as this time point ensured that the wounds in all groups were not fully healed that allowed us to distinguish between inflammatory responses and the healing process. The wounds in all groups were in the initial stage of infection on day 7, and numerous neutrophils represented severe inflammation (Fig. S29). On day 9, H&E staining images showed infiltration of inflammatory cells and acute neutrophils (green arrow) in other groups, while LP@FeG treatment resulted in the proliferation of fibroblasts, well-developed blood vessels, and intact epidermis, accompanied by the emergence of hair follicles in the healing tissue (Fig. 8A). The relative scar width in the LP@FeG group was the smallest at 16.82%, lower than that in other groups (Fig. 8B). Besides, Masson staining indicated a higher degree of newly formed collagen (green arrow) in the LP@FeG group compared to other groups (Fig. 8C and D). LP@FeG accelerated wound healing by reducing inflammatory cells and enhancing collagen deposition, showing marked therapeutic effects in promoting diabetic wound healing in vivo.

To further study diabetic wound regeneration, we conducted immunofluorescence staining for interleukin-6 (IL-6), tumor necrosis factor-α (TNF-α), cluster of differentiation 31 (CD31), α-smooth muscle actin (αSMA), vascular endothelial growth factor (VEGF), CD86, CD206, lymphocyte antigen 6 complex, locus G (Ly6G), and CD3. The proinflammatory cytokines IL-6 and TNF-α served as critical indicators of inflammatory status. CD31 indicated blood vessel regeneration, and αSMA indicated activation of angiogenic responses. In the LP@FeG group, IL-6 and TNF-α expressions were down-regulated, while VEGF expression was up-regulated and the expressions of CD31 (green) and αSMA (pink) were recovered (Fig. 8E), indicating enhanced neovascularization and accumulation of myofibroblast proteins in the wound tissue. M1 macrophages released proinflammatory factors to boost immunity during inflammation, while M2 macrophages released anti-inflammatory factors in the inflammation resolution stage facilitating wound repair. CD86 represented for M1 macrophages, and CD206 represented M2 macrophages. In the subcutaneous layer of wound, there were less CD86 but more CD206 in the LP@FeG group, indicating that LP@FeG induced a shift in macrophage polarization toward the M2 phenotype. In addition, we assessed the expression of Ly6G and CD3 at the wound site. Ly6G served as a marker for neutrophils and reflected the intensity of inflammatory response in the wound area, while CD3 was a T cell marker to evaluate the adaptive immune response at the wound site. After treatment by LP@FeG, the number of Ly6G-positive cells was lower compared with other groups, indicating effective control of the inflammatory response. The number of CD3-positive cells was higher, suggesting an enhanced adaptive immune response and thus enhanced wound healing and repair. These results confirmed the role of LP@FeG in modulating the wound microenvironment, including promoting M2 macrophage polarization, controlling inflammation, and enhancing adaptive immune function. Quantitative analyses indicated that LP@FeG lowered the expression levels of IL-6, TNF-α, CD86, and Ly6G, demonstrating the effectiveness in restraining inflammation and promoting wound healing (Fig. 8F, G, K, and M). The treatment with LP@FeG resulted in enhanced expressions of CD31, αSMA, VEGF, CD206, and CD3 in new granulation tissue (Fig. 8H to J, L, and N). We performed immunohistochemical analysis on day 7 to demonstrate that the wound healing effect resulted from modulation of wound immune environment by LP@FeG (Fig. S30). Collectively, the quantitative results highlighted the dual capability of LP@FeG to suppress inflammatory responses while simultaneously promoting angiogenesis, immune regulation, and tissue regeneration, thereby creating an optimal microenvironment for wound healing (Fig. S31).

To evaluate the changes at different stages of wound healing, we conducted histological studies (Fig. S32). H&E staining showed marked inflammatory cell infiltration on day 3, indicative of the initial inflammatory phase. On day 7, the number of inflammatory cells decreased and granulation tissue formed. On day 12, wounds exhibited substantial reepithelialization and a well-organized tissue structure, reflecting advanced wound maturation (Fig. S33A). Masson staining demonstrated sparse collagen fibers on day 3, an increased collagen deposition on day 7, and a well-organized collagen network on day 12, indicating effective tissue remodeling and repair (Fig. S33B). Immunofluorescence analysis revealed distinct temporal changes in the expression of key biomarkers. IL-6 and TNF-α levels peaked on day 3 and decreased on day 12, reflecting effective resolution of inflammation (Fig. S34). VEGF expression peaked on day 7 and subsequently decreased on day 12, suggesting controlled angiogenesis without pathological neovascularization. αSMA, a marker for myofibroblast activity, was initially low but increased during wound maturation, indicating ongoing tissue remodeling rather than pathological fibrosis. Macrophage polarization analysis revealed a transition from a predominantly proinflammatory (M1 and CD86) state on day 3 to an anti-inflammatory (M2 and CD206) phenotype on day 12, highlighting the shift from inflammation to tissue repair. Neutrophils (Ly6G-positive) were abundant during the early phase but were reduced on day 12, indicating effective control of acute inflammation. In contrast, T cells (CD3-positive) increased on day 9, indicating an active adaptive immune response that supported wound healing during later stages. The quantitative data further confirmed these findings, with marked reductions in IL-6, TNF-α, CD86, and Ly6G levels and marked increases in VEGF, CD31, αSMA, CD206, and CD3 levels over time (Fig. S35). At day 7 posttreatment, LP@FeG promoted a robust cellular response characterized by the rapid proliferation of fibroblasts, marked angiogenesis, intensive collagen deposition, and pronounced immunomodulatory effects, especially macrophage polarization from proinflammatory (M1 and CD86) to anti-inflammatory (M2 and CD206) phenotypes. This early intervention resulted in markedly accelerated tissue regeneration compared to untreated controls. By day 9, diabetic wounds treated with LP@FeG had transitioned from the active proliferation stage to a remodeling stage, characterized by reduced cellular infiltration and the initiation of collagen fiber maturation and remodeling processes. Although histological signs of tissue proliferation (such as fibroblast density and angiogenesis) appeared more vigorous at earlier time points (day 7), day 9 represented the commencement of the tissue maturation stage, where collagen fibers became organized and stabilized but visually appeared less “active” in histological analyses. Collectively, LP@FeG effectively modulated the immune environment, promoted tissue remodeling, and facilitated optimal wound healing beyond simple wound closure. We also measured the hematological parameters including alanine transaminase, aspartate transaminase, creatinine, blood urea nitrogen, white blood cell, red blood cell, and hemoglobin. The blood chemistry results showed that LP@FeG had no adverse effects on mice (Figs. S36 and S37). LP@FeG promoted collagen deposition, reduced inflammation, and enhanced angiogenesis, showing potent therapeutic capability in modulating key biological pathways to accelerate diabetic wound healing (Fig. S38). To evaluate the long-term biosafety of LP@FeG, we conducted histopathological analysis of major organs (heart, liver, spleen, lung, and kidney) and hematological and biochemical analyses. After 30 d, no obvious tissue damage or inflammatory infiltration was observed in H&E-stained sections (Fig. S39). In addition, hematological parameters and blood chemistry results showed that LP@FeG had no adverse effects on mice (Figs. S40 and S41). These results indicate favorable in vivo biosafety of LP@FeG under the treatment regimen.

## Conclusion

In this work, we develop the LP@FeG composite as a therapeutic strategy for diabetic wound management, addressing the dual challenges of hyperglycemia and MDR bacterial infections. For diabetic wound therapy, LP@FeG exerts antibacterial effects through multiple synergistic mechanisms, including the modulation of the wound environment, generation of ROS, and induction of iron-dependent bacterial ferroptosis-like death. Specifically, the metabolic activity of probiotics regulates local glucose levels and pH, thereby creating a microenvironment favorable for nanozyme catalysis, while Fe NZs induce oxidative stress and iron-mediated membrane damage in bacteria. The anti-inflammatory and proangiogenic effects of LP@FeG enhance its therapeutic efficacy, positioning it as a promising approach for diabetic wound care. LP@FeG may be useful for treating other diseases involving MDR bacterial infections, such as burns, bone and joint infections, and gastrointestinal infections. In addition, probiotics and nanozymes can be modified to target other inflammatory diseases caused by bacterial infections, thereby broadening their potential therapeutic applications. The synergistic actions of probiotics and nanozymes make them promising bioactive materials for in vivo catalysis, offering advantages over natural enzymes and conventional therapeutic agents. To ensure its viability for clinical application, the long-term efficacy and safety of LP@FeG in different infection models should be studied in the future.

This work distinguishes itself from previous studies by combining multiple synergistic functionalities into a single therapeutic platform specifically designed for diabetic wound healing. Unlike previous approaches focusing on individual probiotic benefits, ROS generation, or targeted delivery mechanisms, our work integrates LP with FeG to achieve a multifaceted approach. This platform reduces local glucose levels, generates sustained ROS for antibacterial activity, and regulates immune responses and angiogenesis by promoting M2 macrophage polarization, thereby supporting tissue regeneration. Moreover, LP@FeG shows prolonged stability under physiological conditions, minimizing the need for frequent reapplication and reducing systemic toxicity compared to the methods highlighted in previous studies. Collectively, this integrated strategy provides a more comprehensive and effective therapeutic solution, addressing key challenges in chronic diabetic wound management by targeting bacterial elimination, metabolic control, and tissue repair, which have not been concurrently achieved in previous studies.

## Methods

### Synthesis of Fe NZs

Fe NZs were synthesized using a one-step, mild hydrothermal method. *p*-Phthalic acid (18 mg, 0.1 mM), ferric chloride hexahydrate (27 mg, 5 mM), and 400 μl of deionized water were added to 20 ml of absolute ethanol. The mixture was stirred at 1,200 rpm for 10 min and then incubated in water bath at 50 °C for 1 h. After centrifugation at 12,000 rpm for 15 min, the Fe NZs were rinsed with anhydrous ethanol. Finally, the precipitates were dispersed in water for future use.

### Synthesis of FeG

GOx (5 mg) was mixed with the prepared Fe NZs (1 mg/ml, 4 ml) and stirred overnight. FeG was collected by centrifugation at 8,000 rpm for 5 min to remove excess GOx. Finally, FeG was resuspended in water for later use.

### Cultivation of probiotics

Strains of LP, *S. cerevisiae*, *B. subtilis*, *B. cereus*, *B. licheniformis*, and *E. coli* Nissle 1917 were retrieved from an −80 °C freezer and inoculated onto solid agar plates. After incubation for 24 h, a selected colony was transferred to fresh liquid culture for incubation overnight. Finally, the probiotics were washed and suspended in PBS to prepare the stock solution (1 × 10^8^ CFU/ml).

### Characterizations

TEM and SEM images were captured using a JEOL 2100Plus TEM and a GeminiSEM 300, each at an accelerating voltage of 120 kV. HAADF-scanning TEM–energy-dispersive x-ray spectroscopy was acquired using a Talos F200X electron microscope (Thermo Fisher Scientific, USA). Zeta potential was measured by dynamic light scattering with a Zeta Sizer Nano ZS90 (Malvern, UK). Crystal structures were analyzed over the range of 5° to 80° using XRD (Rigaku SmartLab SE, Japan) with high-intensity Cu Kα radiation. Chemical compositions were determined using XPS (Thermo Fisher Scientific K-Alpha, USA). Absorbance was recorded using an Infinite 200Pro (TECAN, Switzerland). The pH value was determined using a pH meter (WTW, Germany). Live/dead staining was visualized using fluorescence microscopy (DMi8, Leica, Germany). Blood glucose levels were measured using blood glucose meter (Yuyue, China). H&E staining was observed using a microscope (DM4B, Leica, Germany). Immunofluorescence staining was performed using a Nikon A1 LSCM (Nikon, Japan).

### POD-like activity of Fe NZs

TMB was used as the substrate to investigate the POD-like activity of Fe NZs. Briefly, 10 μl of Fe NZs (100 μg/ml) and 20 μl of TMB (5 mM) were mixed into a sodium acetate buffer (pH 4.0) containing 10 μl of H_2_O_2_ (4 mM). UV–vis spectroscopy was used to measure the absorbance at 652 nm to determine the POD-like activity of Fe NZs. The steady-state kinetics of Fe NZs were monitored using a microplate reader.

### Glucose metabolism assessment

The glucose concentration was measured using the 3,5-dinitrosalicylic acid method. Specifically, 500 μl of the solution from each time point was withdrawn and mixed with 1.5 ml of 3,5-dinitrosalicylic acid. The mixture was then heated for 5 min at 100 °C and transferred to an ice bath for 20 min. Subsequently, the glucose concentration was determined by measuring the absorbance at 540 nm.

### pH assessment

The pH value was monitored at each time point using a laboratory-grade pH meter involving glucose (5 mM) and LP@FeG (50 μg/ml).

### Cascade catalytic reactions mediated by LP@FeG

LP@FeG was incubated with glucose solutions at various concentrations. TMB was then introduced to the aforementioned mixture. The catalytic activity of LP@FeG was evaluated by monitoring the absorbance of oxidized TMB at 652 nm using a microplate reader.

### Bacterial viability assessment

LP (1 × 10^7^ CFU/ml) and LP@FeG (50 μg/ml) were incubated at 37 °C for 2 h in PBS buffer (pH 7.4). Bacterial counts of LP were quantified using the plate counting method.

### Antibacterial efficacy of LP@FeG

CREC and MRSA were washed with sterile PBS and categorized into 7 groups: (a) PBS, (b) glucose, (c) glucose + Fe NZs, (d) FeG, (e) glucose + FeG, (f) glucose + LP, and (g) glucose + LP@FeG. The concentrations of bacteria, glucose, and LP@FeG were maintained at 10^6^ CFU/ml, 5 mM, and 50 μg/ml, respectively. The suspensions were incubated at 37 °C for 2 h. After incubation, 100 μl of suspension was spread onto LB agar plates, incubated for 48 h at 37 °C, and analyzed using ImageJ.

### Microbial morphology assessment

The structural characteristics of LP, CREC, and MRSA were analyzed using field emission SEM. Bacteria were fixed at 4 °C using 2.5% glutaraldehyde solution overnight, rinsed with PBS buffer, and then fixed in a 1% osmium tetroxide solution for 1 to 2 h. Following this, bacteria were subjected to graduated ethanol dehydration series, involving solutions of increasing concentration (30%, 50%, 70%, 90%, and 100%), each for 15 min. The dehydrated samples were dried and observed using a Hitachi SU-8010 scanning electron microscope.

### Staining of live and dead cells for LP, CREC, and MRSA

Samples of LP, CREC, and MRSA were rinsed with sterile PBS. Subsequently, the samples were labeled with calcein-acetoxymethyl and propidium iodide dyes and incubated at 37 °C for 30 min, thereby enabling observation using confocal fluorescence microscopy.

### Nucleic acid, protein, and K^+^ leakage assay

The bacterial suspensions were treated with different groups: (a) PBS, (b) glucose, (c) glucose + Fe NZs, (d) FeG, (e) glucose + FeG, (f) glucose + LP, and (g) glucose + LP@FeG. The nucleic acid leakage was quantified by measuring the absorbance at 260 nm. The protein leakage from bacterial cells was assessed using the enhanced BCA Protein Assay Kit (Beyotime). The K^+^ concentration was quantified using the Potassium (K) Turbidimetric Assay Kit.

### ATP level, DNA damage, and lipid POD assay

Bacteria underwent the same treatment as described above and were then centrifuged to remove the supernatant. The ATP level was determined using the ATP Assay Kit (S0026, Beyotime). The DNA damage was assessed using the Apoptosis Detection Kit (C1086, Beyotime). Lipid peroxidation levels were quantified with a malondialdehyde content assay kit (BC0025, Solarbio).

### Ferroptosis in CREC and MRSA

To evaluate the effect of iron inhibitors on the bactericidal activity of LP@FeG, various concentrations (0, 100, 200, 300, 400, and 500 mM) of ATP, l-Glu, GSH, and VC were incubated with treated CREC/MRSA. Bacterial counts were quantified after 24 h of incubation at 37 °C using the plate counting method. The malondialdehyde assay kit (Solarbio, Beijing, China) was used to determine lipid peroxidation levels in CREC/MRSA.

### GSH depletion test

The GSH and GSSG content was assessed using GSH and GSSG assay kit (S0053, Beyotime). Briefly, the bacterial solutions of CREC/MRSA (10^7^ CFU/ml) were with various treatments at 37 °C. The samples were subjected to protein removal and ultrasonic pulverization. Then, the supernatants were collected and diluted for the determination of GSH and GSSG levels.

### Measurement of bacterial lipid peroxides by BODIPY 581/591 C11

The lipid peroxides of bacteria were measured using BODIPY 581/591 C11 staining (ajci64572, Amgicam). Briefly, bacterial cells were stained with BODIPY C11 (5 μM) for 30 min at 37 °C in darkness. The cells were then washed with PBS and observed using confocal laser scanning microscopy. Furthermore, quantitative analysis of fluorescence intensity was conducted using a multifunctional microplate reader.

### Transcriptome analysis

For transcriptome analysis, MRSA suspension (6 ml, 1 × 10^8^ CFU/ml) was divided into control and LP@FeG groups. After treatment, MRSA suspension was centrifuged at 4,000 rpm for 5 min, washed with PBS, and transferred to dry ice for RNA extraction. Subsequent analyses were carried out by Novogene Science and Technology Corporation (Beijing, China).

### In vitro cytotoxicity assessment

HUVECs, RAW 264.7 cells, and NIH/3T3 cells were suspended and seeded into a 96-well plate. After a 4-h incubation for cell adhesion, the culture medium was removed and replaced with fresh medium. To assess the cytotoxic effects, a CCK-8 assay was utilized. Initially, cells (1.0 × 10^4^ cells per well) were allowed to adhere for 6 h. After incubation for 24 h with various concentrations of LP@FeG, the cells were rinsed with PBS and incubated with CCK-8 solution. The absorbance was subsequently quantified at 450 nm.

### Hemolysis assay

To assess hemocompatibility, mouse blood samples were collected and centrifuged (2,500*g* for 20 min) to isolate red blood cells. The erythrocytes were then washed with PBS and subjected to various treatments. After incubating at 37 °C for 2 h, the samples were centrifuged, and hemocompatibility was evaluated by measuring absorbance at 541 nm of the supernatant.

### Wound healing experiments in diabetic mice

All animal experiments were approved by the Animal Ethics Committee of Hangzhou Medical College (Hangzhou, China). Mouse diabetes models were established in 6- to 8-week-old male mice (20 to 30 g) via intraperitoneal injection of streptozotocin (80 mg/kg). First, the mice were fasted for 16 h. Blood glucose levels were then randomly measured from the tail vein using a glucometer, followed by an intraperitoneal injection of 1% streptozotocin. Blood glucose concentration was recorded every 3 d, with levels exceeding 16.7 mmol/l indicated the successful establishment of the diabetes model. A full-thickness skin wound with a diameter of 8 mm was created on the back of each mouse. The wound was infected with 100 μl of MRSA solution (1 × 10^8^ CFU/ml). After 24 h, mice were divided into 6 treatment groups: (a) control, (b) Fe NZs, (c) FeG, (d) LP, (e) LP@FeG, and (f) vancomycin. For the administration of different materials, 50 μl of each solution was applied to the wounds in the infectious diabetic wound model. After the diabetic wound infection, the treatment was applied every 2 d to ensure effective exposure throughout the healing period. Images of wounds in each group were captured on days 0, 3, 7, and 9. Wounds were observed, measured, and analyzed using ImageJ. Bacterial counts at wound sites were conducted to evaluate the antibacterial effect of LP@FeG. The diluted solution was poured onto the LB agar plates and incubated for 24 h at 37 °C. The colonies on the plate were then counted. On the last day, all mice were euthanized, and tissues from wounds, heart, lung, spleen, liver, kidney, and blood were collected for further examination. The collected tissues were immersed in 4% paraformaldehyde and fixed for 48 h. Subsequently, the tissues were prepared for histological analysis, including H&E staining, Masson staining, IL-6 staining, TNF-α staining, CD31/αSMA staining, VEGF staining, CD86/CD206 staining, Ly6G staining, and CD3 staining.

### Statistics and data analyses

All statistical analyses were performed by GraphPad Prism 9 (GraphPad Software). The statistical significance was indicated as *P < 0.05; **P < 0.01; ***P < 0.001; ****P < 0.0001; n.s., no significance.

## Data Availability

The authors state that the data supporting the findings of this study are provided in the main article and the Supplementary Materials and may also be obtained from the corresponding authors upon reasonable request.

## Supplementary Materials

Supplementary 1 Chemicals and Reagents Figs. S1 to S42 Table S1
