# Supplementary material for: Nanozymes-Armed Probiotic Lactobacillus plantarum Coordinates Ferroptosis-like Bacterial Death for Diabetic Wound Therapy
Source: Research (Wash D C). 2026 May 21;9:1258. doi: 10.34133/research.1258 (PMC13191088; doi:10.34133/research.1258)
Supplement: Supplementary 1 — Chemicals and Reagents Figs. S1 to S42 Table S1 [file research.1258.f1.docx]

Supporting Information for

**Nanozymes-armed probiotic *Lactobacillus plantarum* coordinates ferroptosis-like bacterial death for diabetic wound therapy**

Jinjie Hou ^a,b^, Ruijie Fu ^a,b^, Mengliu Zhao ^a,b^, Anlai Zou ^a,b^, Yidan Wang ^a,b^, Zexiang Wang ^a,b^, and Yunlei Xianyu ^a, b, *^

^a^ Department of Clinical Laboratory of Sir Run Run Shaw Hospital, College of Biosystems Engineering and Food Science, Zhejiang University School of Medicine, Hangzhou 310058, People’s Republic of China

^b^ Key Laboratory of Precision Medicine in Diagnosis and Monitoring Research of Zhejiang Province, Sir Run Run Shaw Hospital, Hangzhou 310016, People’s Republic of China

^*^ Corresponding Author.

E-mail: xianyu19@zju.edu.cn.

**Content List**

[Chemicals and Reagents 4](#_Toc219291019)

[Supplementary Figs 5](#_Toc219291020)

[Fig.S1. 5](#_Toc219291021)

[Fig.S2 6](#_Toc219291022)

[Fig.S3 7](#_Toc219291023)

[Fig.S4 8](#_Toc219291024)

[Fig.S5 9](#_Toc219291025)

[Fig.S6 10](#_Toc219291026)

[Fig.S7 11](#_Toc219291027)

[Fig.S8 12](#_Toc219291028)

[Fig.S9 13](#_Toc219291029)

[Fig.S10 14](#_Toc219291030)

[Fig.S11. 15](#_Toc219291031)

[Fig.S12. 16](#_Toc219291032)

[Fig.S13 17](#_Toc219291033)

[Fig.S14 18](#_Toc219291034)

[Fig.S15. 19](#_Toc219291035)

[Fig.S16 20](#_Toc219291036)

[Fig.S17 21](#_Toc219291037)

[Fig.S18. 22](#_Toc219291038)

[Fig.S19 23](#_Toc219291039)

[Fig.S20 24](#_Toc219291040)

[Fig.S21 25](#_Toc219291041)

[Fig.S22 26](#_Toc219291042)

[Fig.S23 27](#_Toc219291043)

[Fig.S24 28](#_Toc219291044)

[Fig.S25 29](#_Toc219291045)

[Fig.S26 30](#_Toc219291046)

[Fig.S27 31](#_Toc219291047)

[Fig.S28 32](#_Toc219291048)

[Fig.S29. 33](#_Toc219291049)

[Fig.S30. 34](#_Toc219291050)

[Fig.S31. 35](#_Toc219291051)

[Fig.S32. 36](#_Toc219291052)

[Fig.S33. 37](#_Toc219291053)

[Fig.S34. 38](#_Toc219291054)

[Fig.S35. 39](#_Toc219291055)

[Fig.S36 40](#_Toc219291056)

[Fig.S37 41](#_Toc219291057)

[Fig.S38 42](#_Toc219291058)

[Fig.S39 43](#_Toc219291056)

[Fig.S40 44](#_Toc219291057)

[Fig.S41 45](#_Toc219291058)

[Table S1. 46](#_Toc219291059)

# **Chemicals and Reagents**

Ferric chloride hexahydrate (FeCl_3_·6H_2_O), hydrogen peroxide (H_2_O_2_), ethylene diamine tetraacetic acid (EDTA), L-glutamic acid (L-Glu), glutathione (GSH), vitamin C (VC), terephthalic acid (TA), and 3,3',5,5'-tetramethylbenzidine (TMB) were purchased from Aladdin Reagents (Shanghai, China). Glucose oxidase (GOx), 3,5-dinitrosalicylic acid (DNS), and anhydrous ethanol were purchased from Macklin (Shanghai, China). Lennox broth (LB) was purchased from Hope Bio-Technology Co., Ltd (Qingdao, China). CCK-8 was provided by Dojindo (Kumamoto, Japan). Reactive oxygen species assay kit and malondialdehyde (MDA) colorimetric assay kit were purchased from Solarbio Science & Technology Co., Ltd (Beijing, China). BCA protein assay kit and ATP assay kit were purchased from Beyotime Biotech Inc (Shanghai, China). Calcein-AM/PI double stain kit was purchased from YEASEN Biotech Co., Ltd (Shanghai, China). All other reagents were purchased from domestic providers and used as received.

# **Supplementary Figs**

**
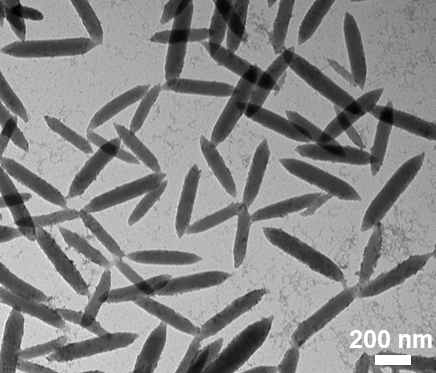
**

Fig.S1. TEM image of FeG.

**
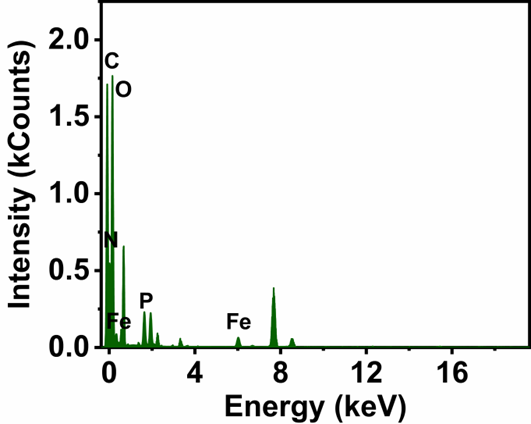
**

Fig.S2**.** EDS spectrum of LP@FeG.


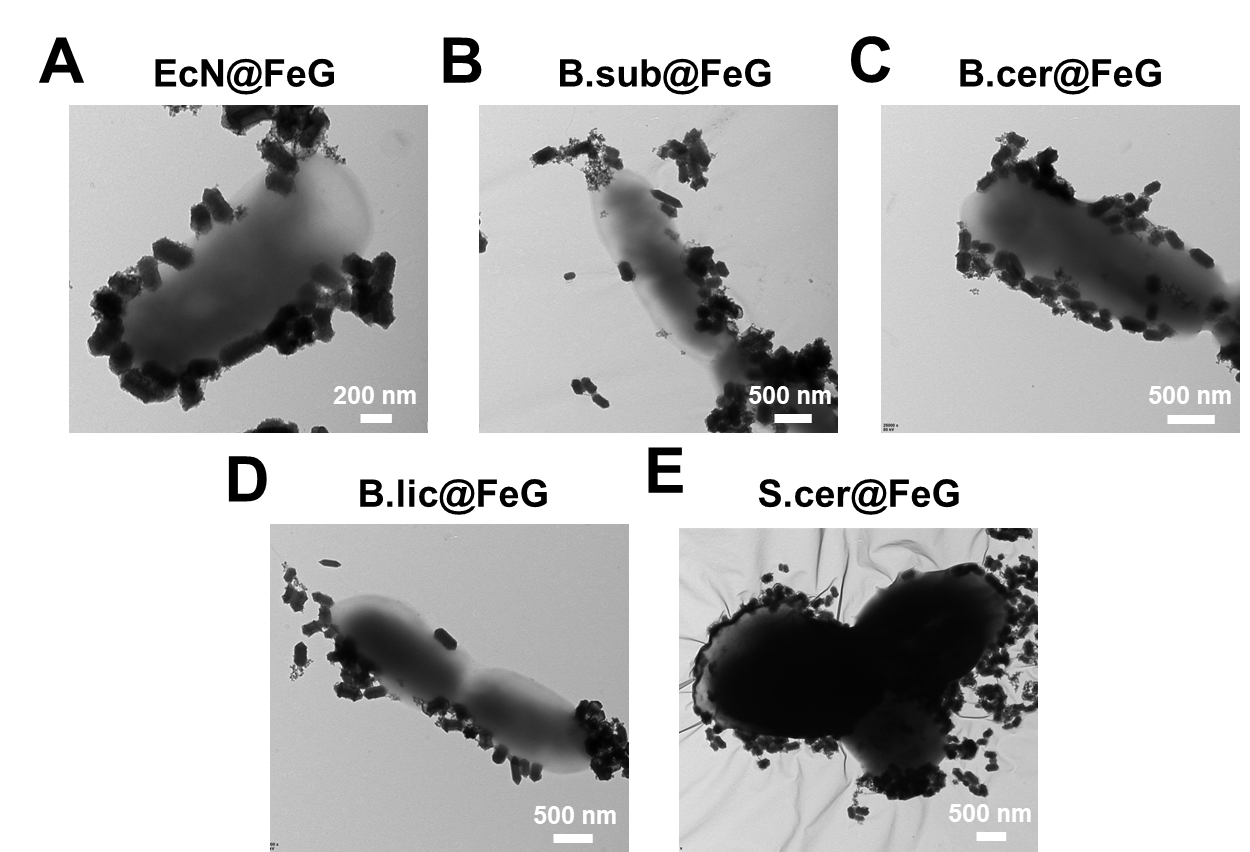


Fig.S3**.** TEM images of probiotics@FeG including (A) EcN@FeG, (B) B.sub@FeG, (C) B.cer@FeG, (D) B.lic@FeG, and (E) S.cer@FeG.

**
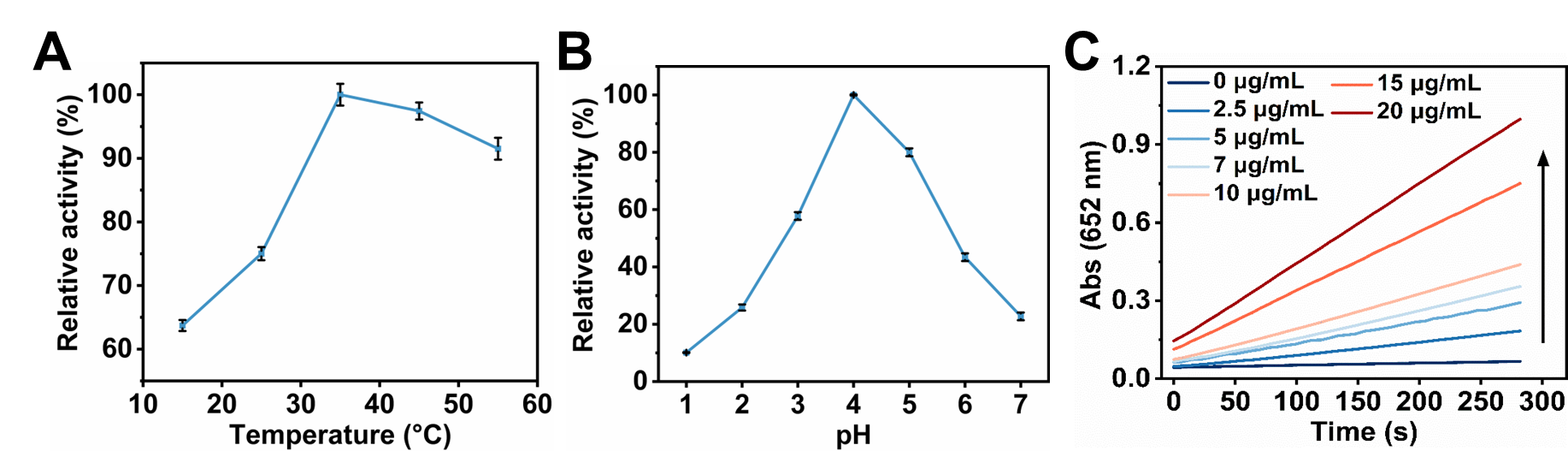
**

Fig.S4**.** Effects of (A) temperature and (B) pH on POD-like activity of Fe NZs. (C) Time-dependent absorbance changes at 652 nm of Fe NZs at different concentrations.


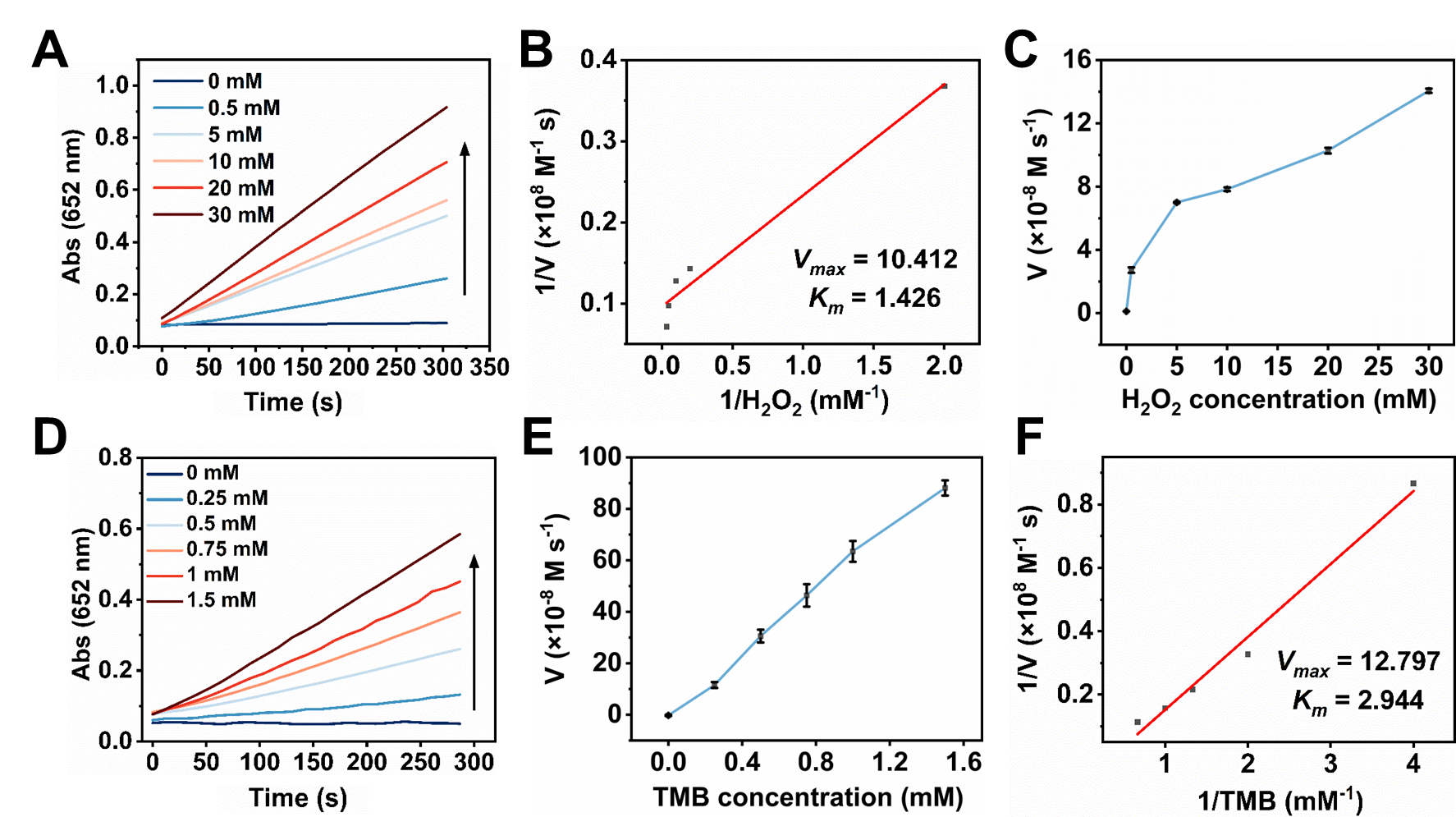


Fig.S5**.** Steady-state kinetics of the POD-like activity of Fe NZs. (A) Time-dependent absorbance of Fe NZs and H_2_O_2_ at different concentrations. (B) Michaelis-Menten curve and (C) Lineweaver-Burk plotting for Fe NZs with TMB as a substrate in the presence of H_2_O_2_. (D) Time-dependent absorbance of Fe NZs and TMB at different concentrations. (E) Michaelis-Menten curve and (F) Lineweaver-Burk plotting for Fe NZs with H_2_O_2_ as a substrate in the presence of TMB.


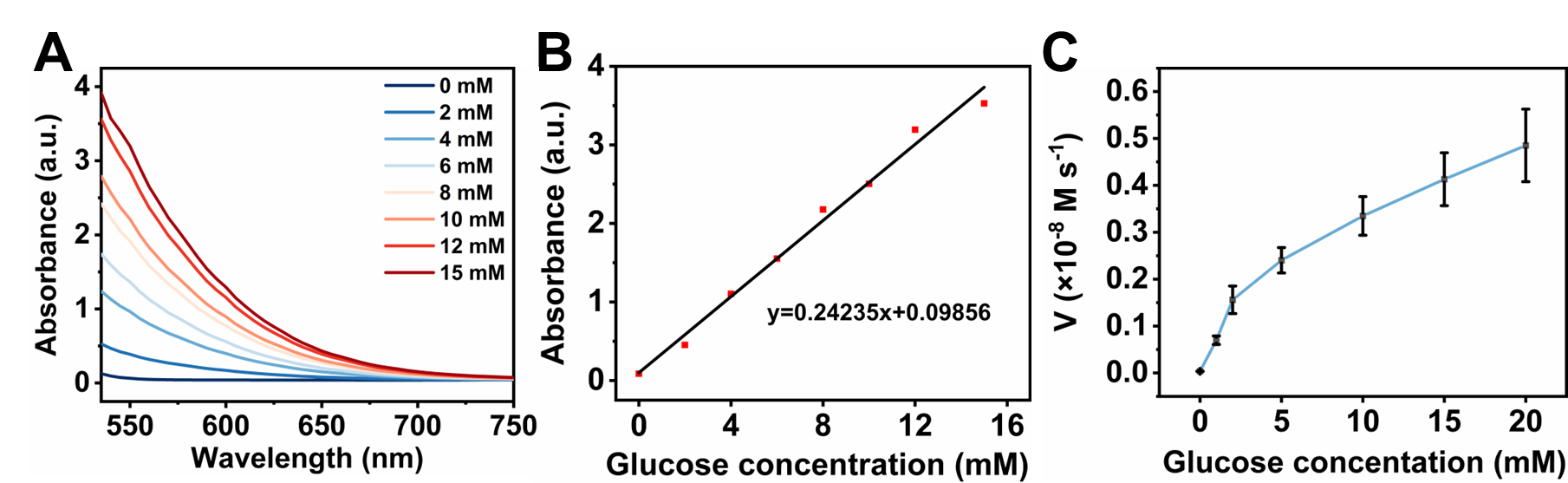


Fig.S6**.** Steady-state kinetics of GOx activity of FeG. (A) Absorbance changes of glucose at different concentrations. (B) Standard curve for quantification of glucose concentrations. (C) Michaelis-Menten kinetic assay for FeG with glucose as a substrate.


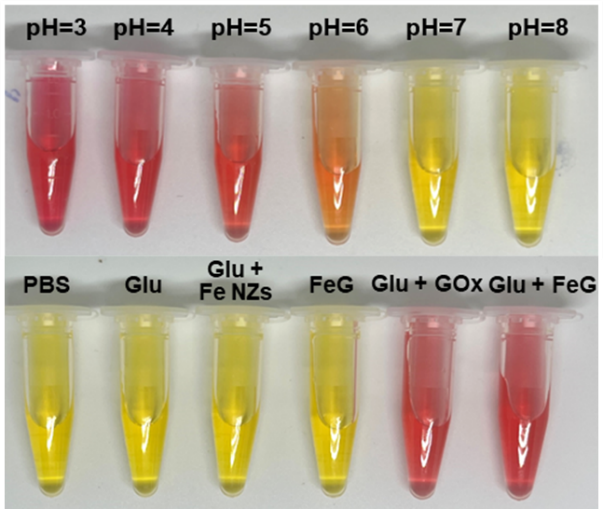


Fig.S7**.** Photographs of different treatments after the addition of 0.001% methyl red.


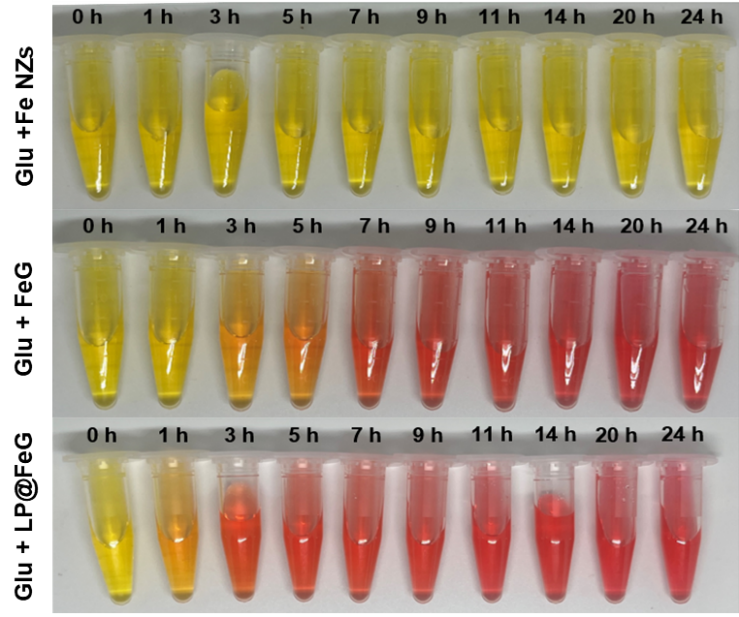


Fig.S8**.** Photographs of different treatments including glucose + Fe NZs, glucose + FeG, and glucose + LP@FeG in PBS buffer (pH 7.4).

**
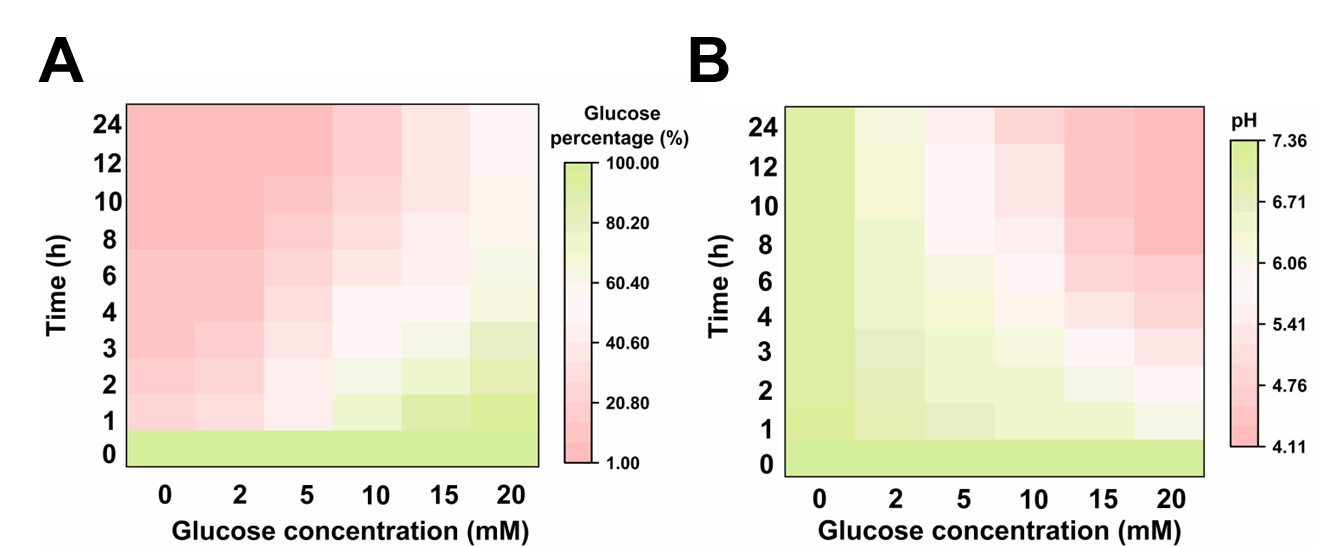
**

Fig.S9**.** Heatmaps showing the variation in (A) glucose concentration and (B) pH for glucose at different concentrations.


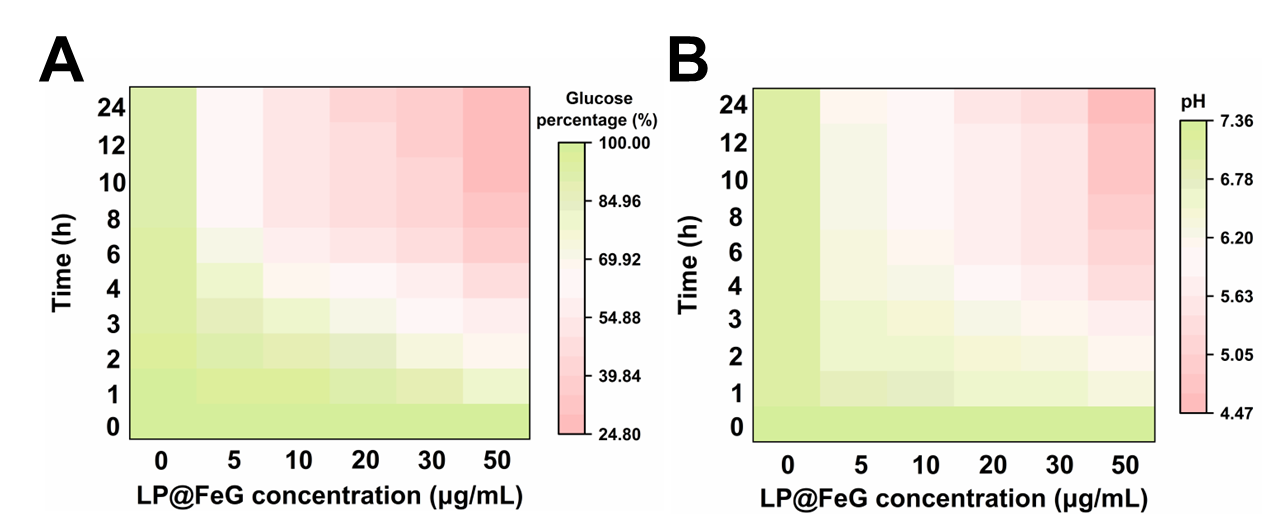


Fig.S10**.** Heatmaps showing the variation in (A) glucose concentration and (B) pH for LP@FeG at different concentrations.


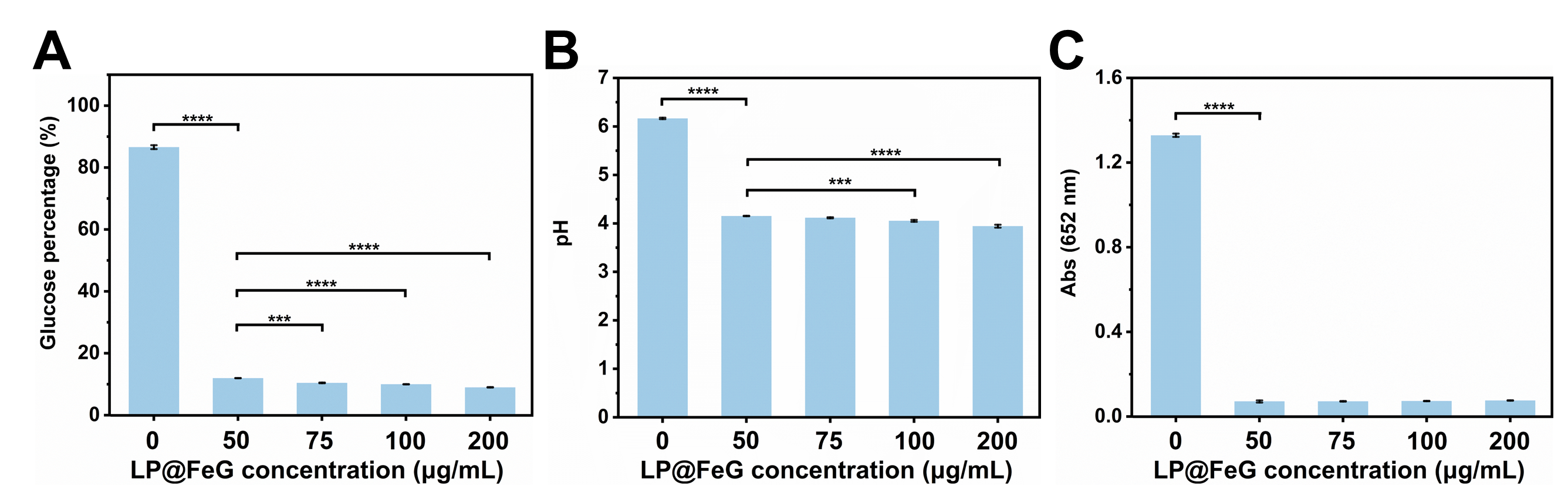


Fig.S11. Effects of different LP@FeG concentrations on glucose consumption, pH reduction, POD-like activity, and antibacterial efficacy. (A) Glucose percentage remaining and (B) pH after treatment with LP@FeG at different concentrations. (C) POD-like activity after treatment with LP@FeG at different concentrations.


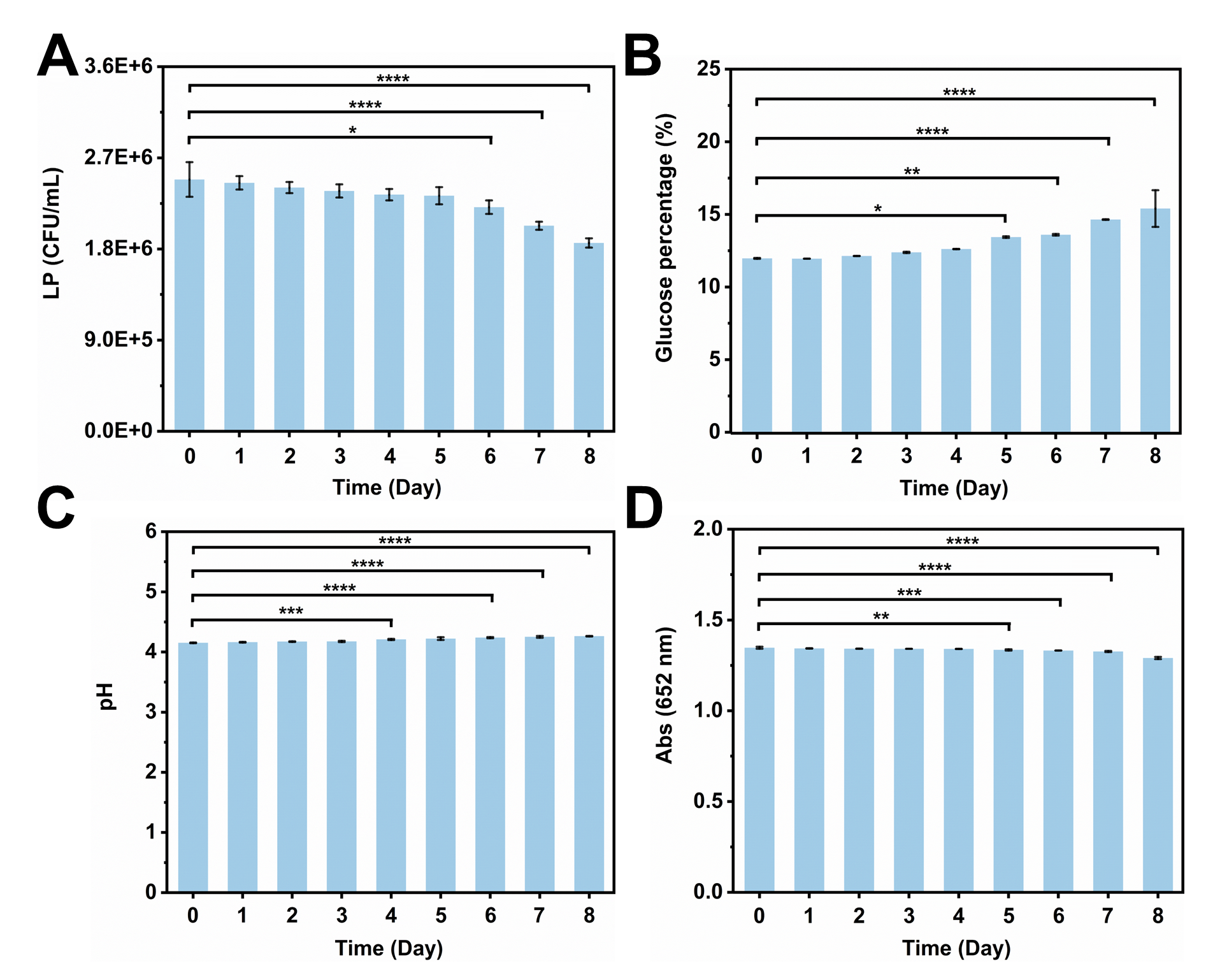


Fig.S12. Stability of LP@FeG over 8-day storage period. (A) Viability of LP in LP@FeG across different time points. (B) Glucose percentage changes during storage. (C) pH value monitoring during storage. (D) Absorbance at 652 nm representing POD-like activity of LP@FeG during storage.

**
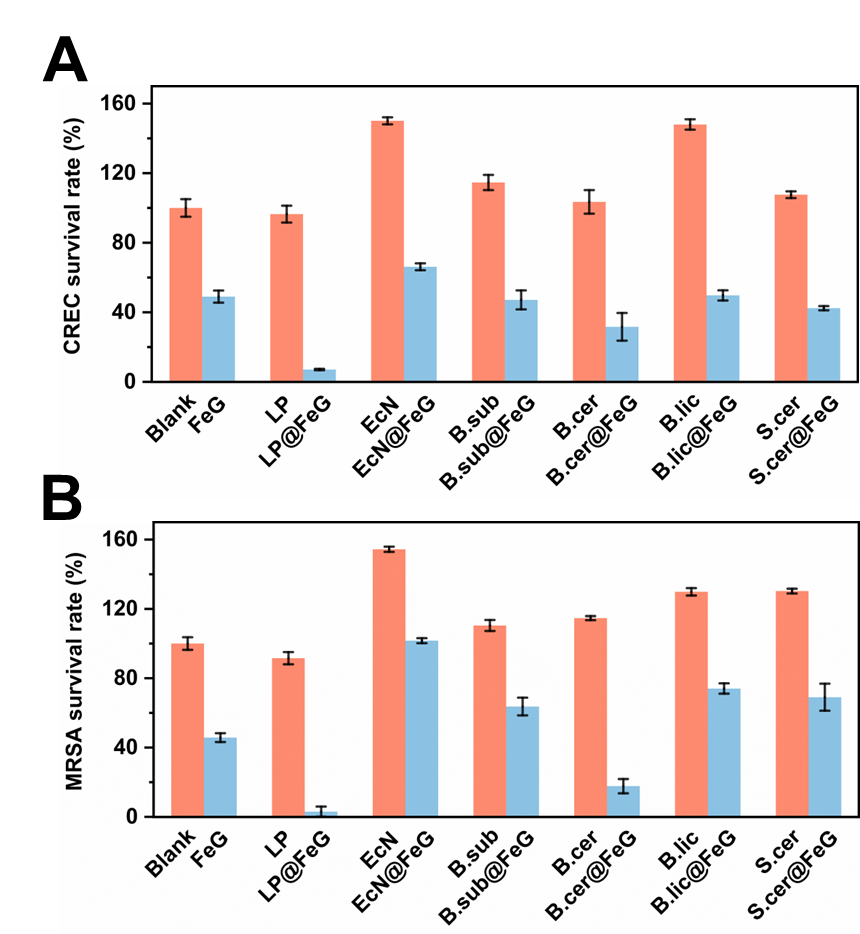
**

Fig.S13**.** Antibacterial effects of probiotics@FeG against (A) CREC and (B) MRSA.


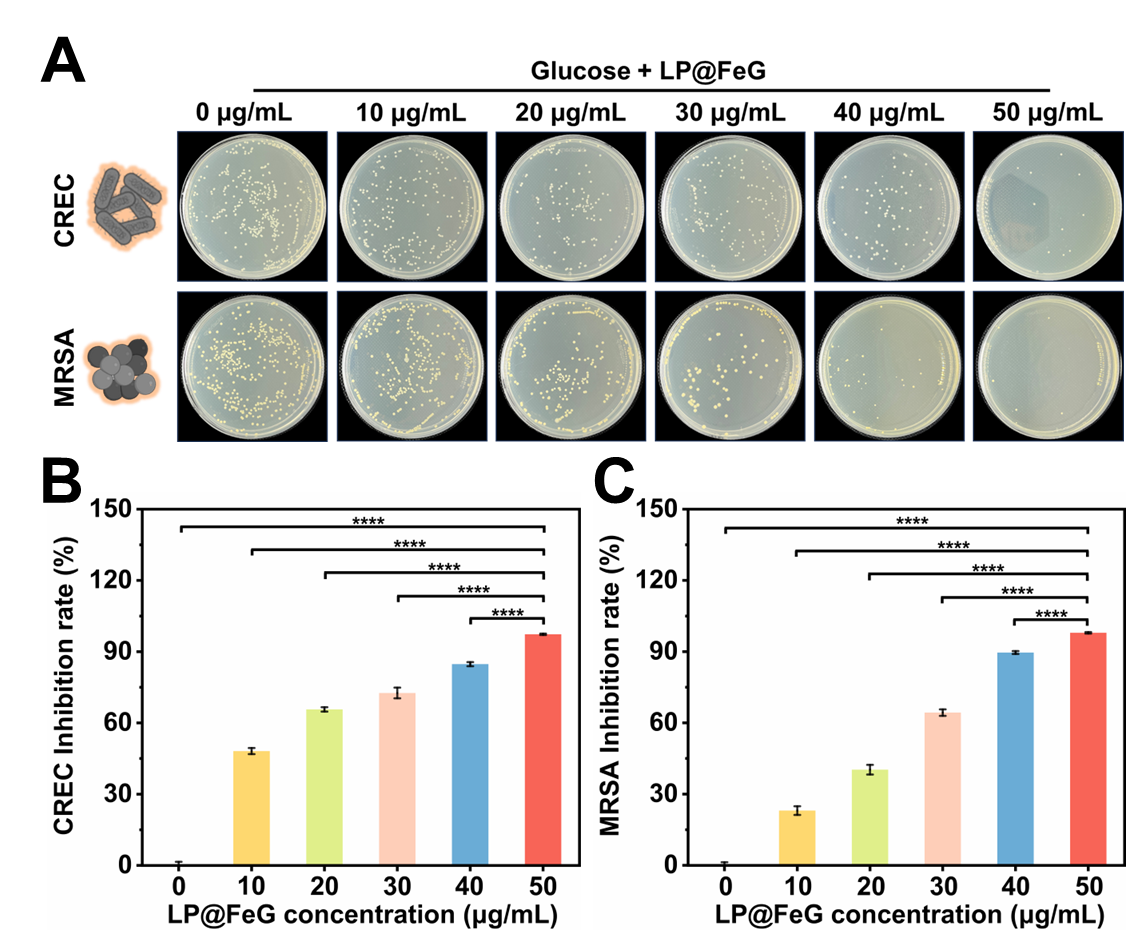


Fig.S14**.** (A) Digital images of CREC and MRSA colonies after the treatment of LP@FeG at different concentrations. Antibacterial effects of LP@FeG at different concentrations against (B) CREC and (C) MRSA.





Fig.S15. Bacterial inhibition rates of LP@FeG at various concentrations against CREC and MRSA.


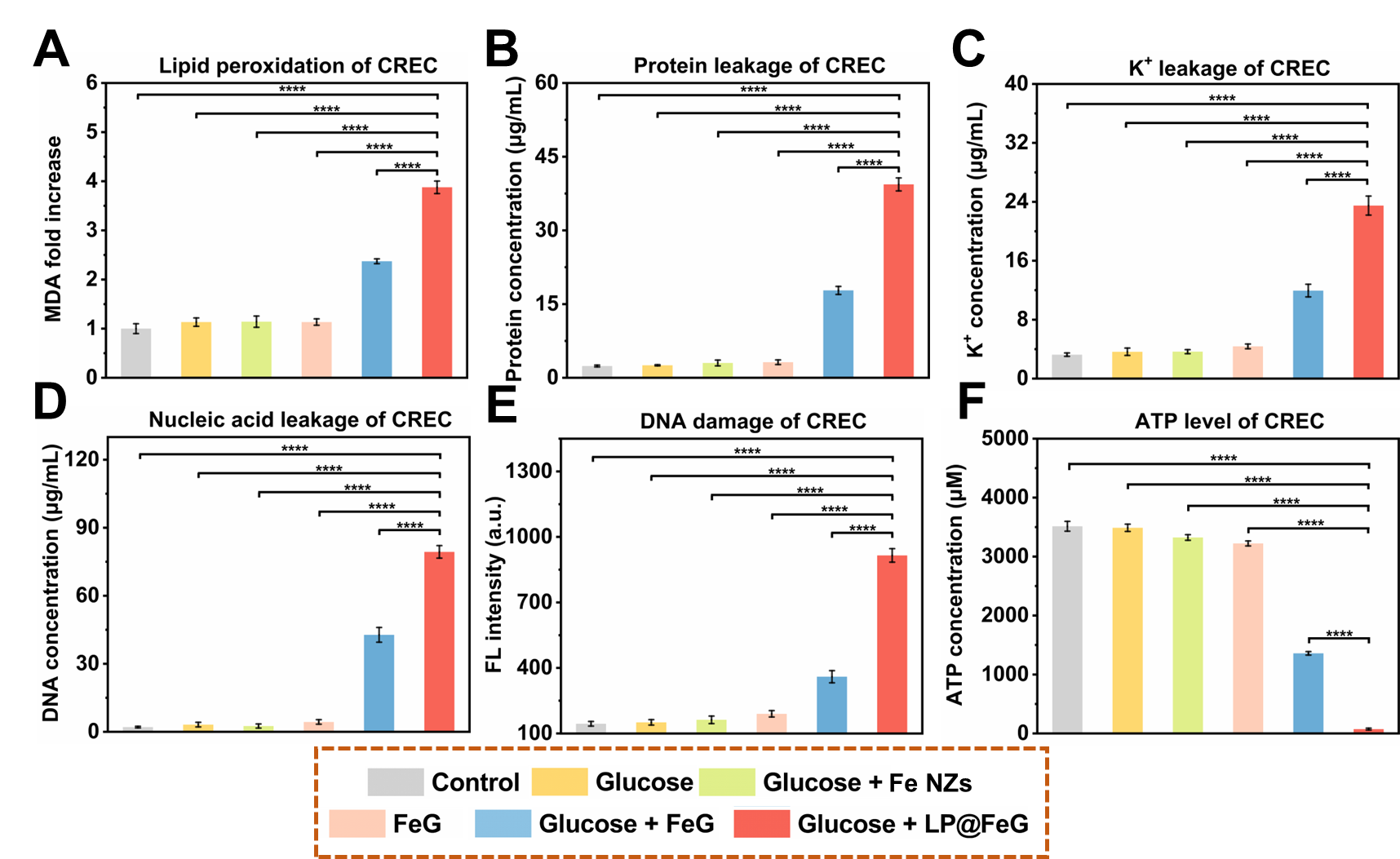


Fig.S16**.** Quantitative analyses of (A) lipid peroxidation, (B) protein leakage, (C) k^+^ leakage, (D) nucleic acid leakage, (E) DNA damage, and (F) ATP level of CREC.


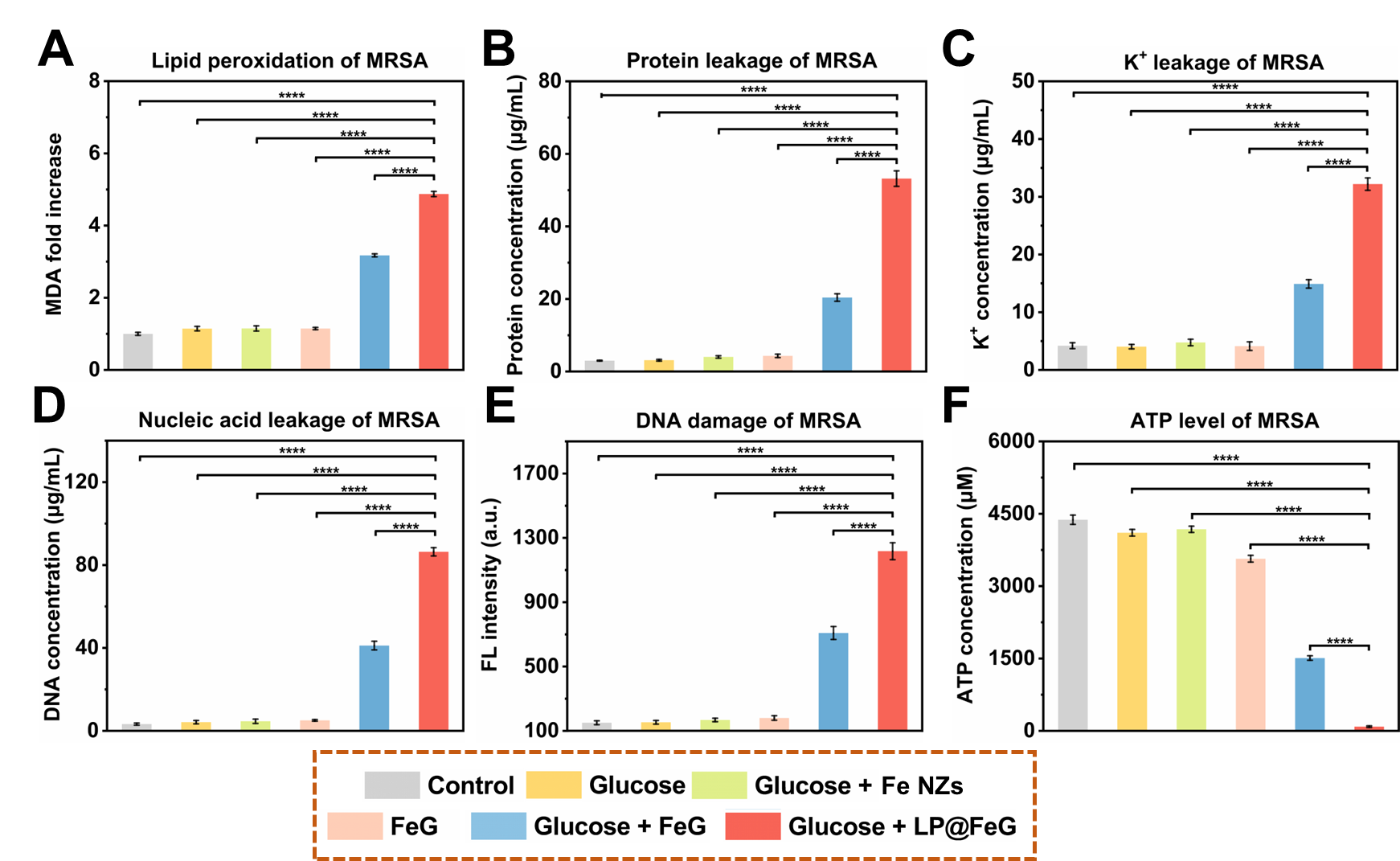


Fig.S17**.** Quantitative analyses of (A) lipid peroxidation, (B) protein leakage, (C) k^+^ leakage, (D) nucleic acid leakage, (E) DNA damage, and (F) ATP level of MRSA.


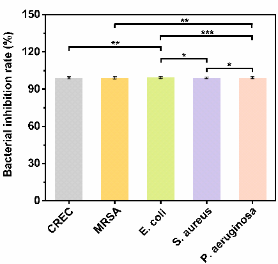


Fig.S18. Bacterial inhibition rate of LP@FeG against various bacterial strains (CREC, MRSA, *E. coli*, *S. aureus*, and *P. aeruginosa*).


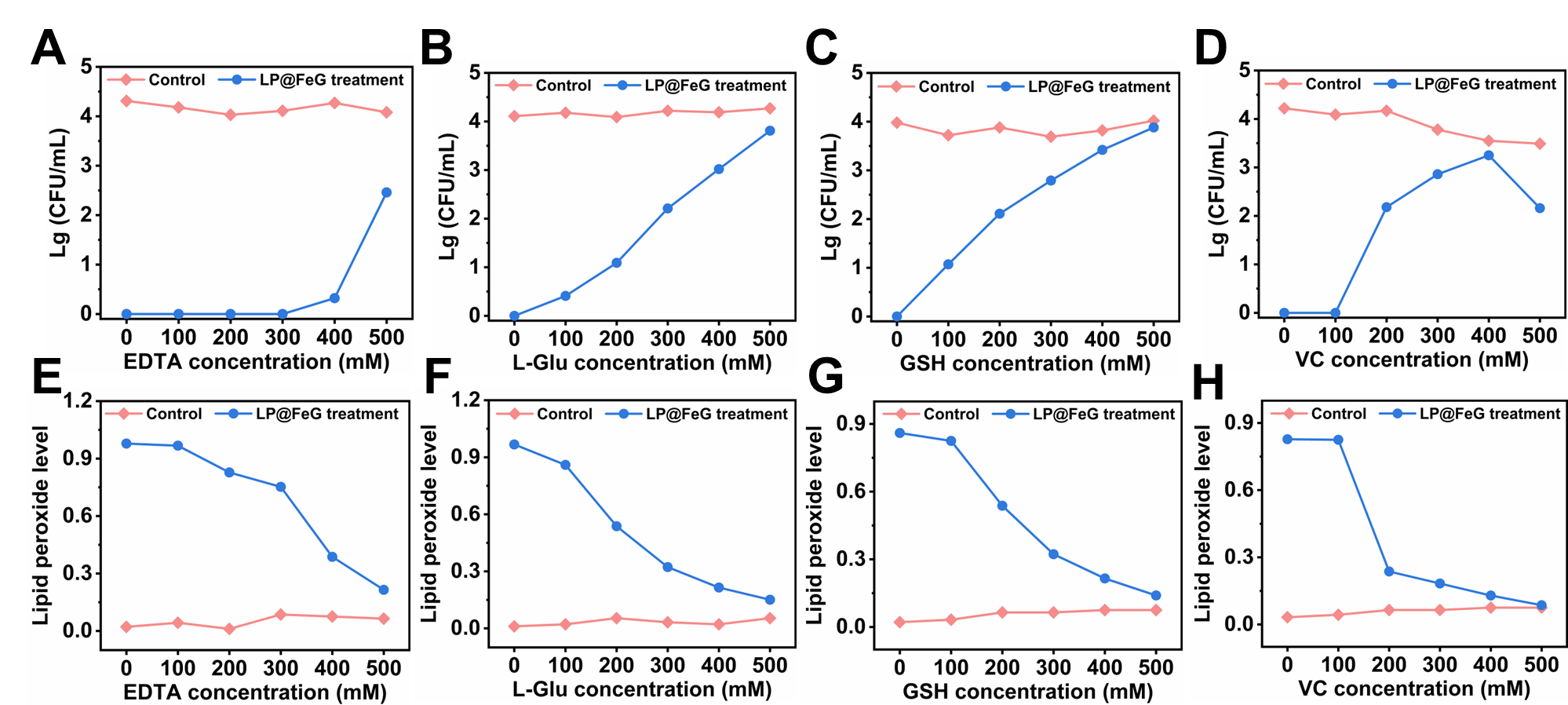


Fig.S19**.** Inhibition effects of (A) EDTA, (B) L-Glu, (C) GSH, and (D) VC on CREC colonies treated by LP@FeG. (E-H) Lipid peroxidation of CREC with different treatments.


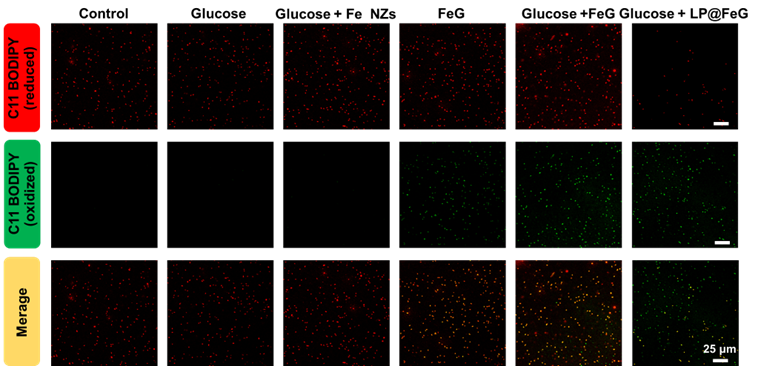


Fig.S20**.** Monitoring of lipid peroxidation in CREC using BODIPY581/591-C11.


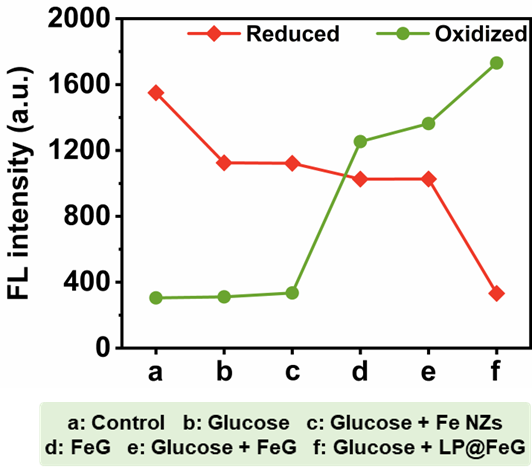


Fig.S21**.** Fluorescence quantification of lipid peroxidation in CREC.





Fig.S22**.** Fe^2+^ concentration in CREC with different treatments.


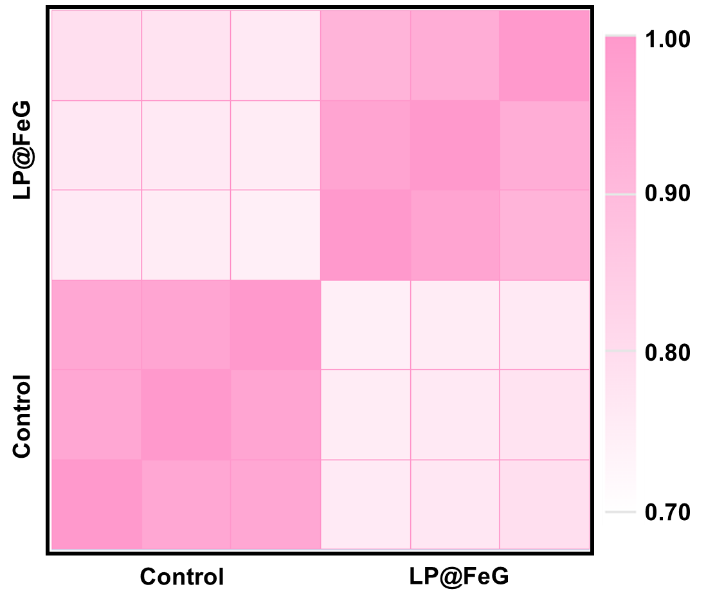


Fig.S23**.** Correlation graph of gene expression between the control and LP@FeG groups.


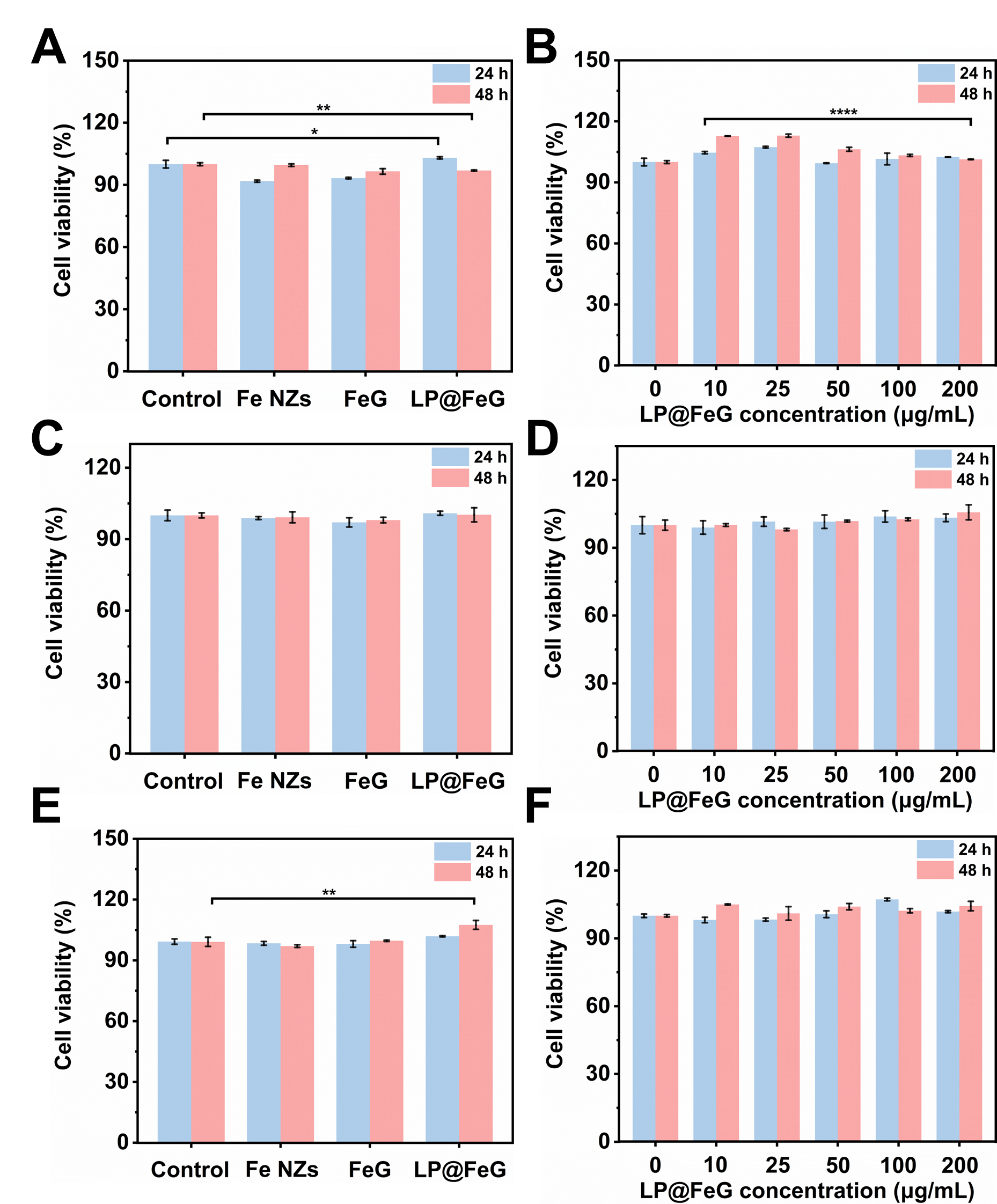


Fig.S24**.** (A, C, E) Cell viability of HUVECs, RAW 264.7 cells, and NIH/3T3 cells in different groups. (B, D, F) Cell viability of HUVECs, RAW 264.7 cells, and NIH/3T3 cells after the treatment of LP@FeG at different concentrations.





Fig.S25**.** Hemolysis test of water, PBS, Fe NZs, FeG, LP, and LP@FeG.





Fig.S26**.** Blood glucose levels in mice in different groups.





Fig.S27**.** Bacterial numbers of MRSA at the wound site on Day 9.





Fig.S28**.** Body weight change of mice in different groups.


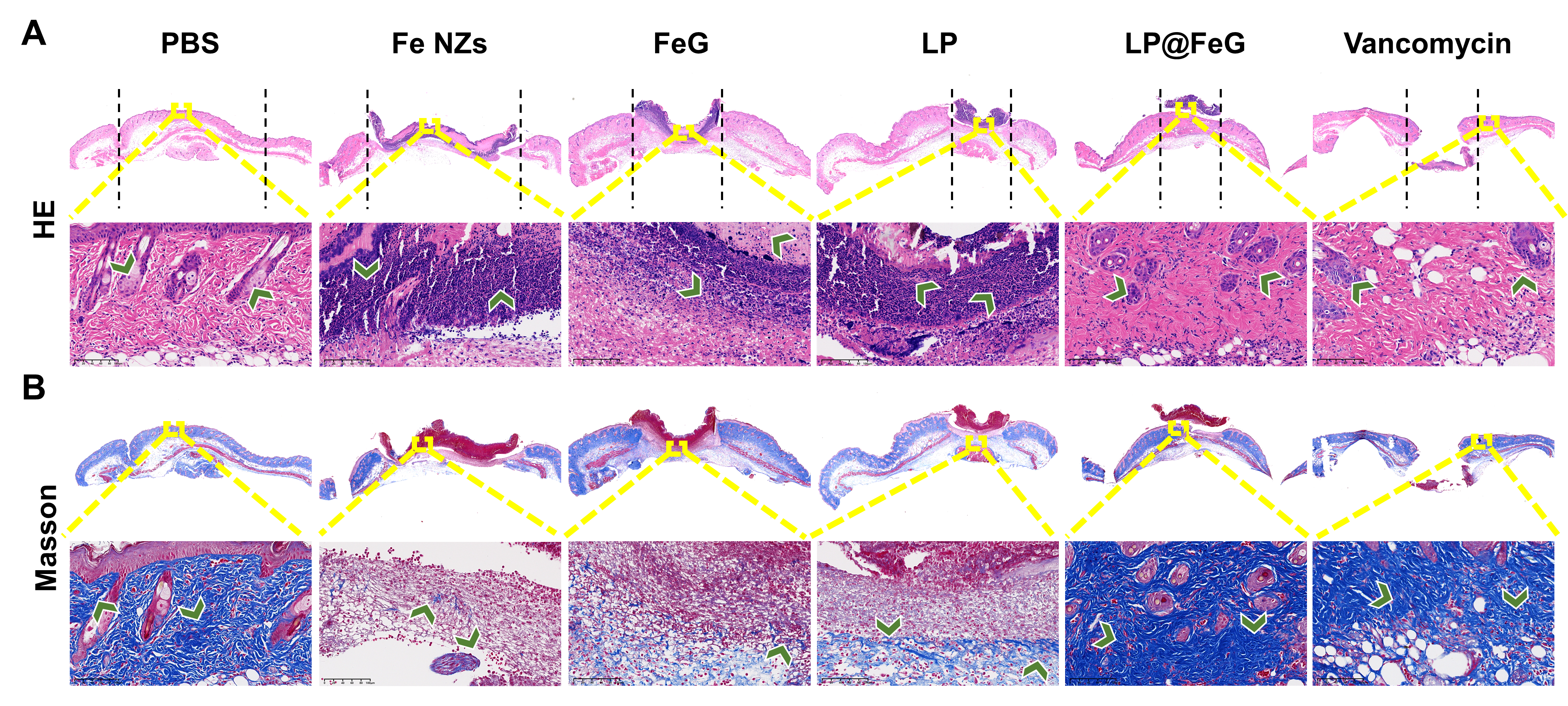


Fig.S29. (A) H&E images of wound tissues in different groups on Day 7. (B) Masson staining images of wound tissues in different groups on Day 7.


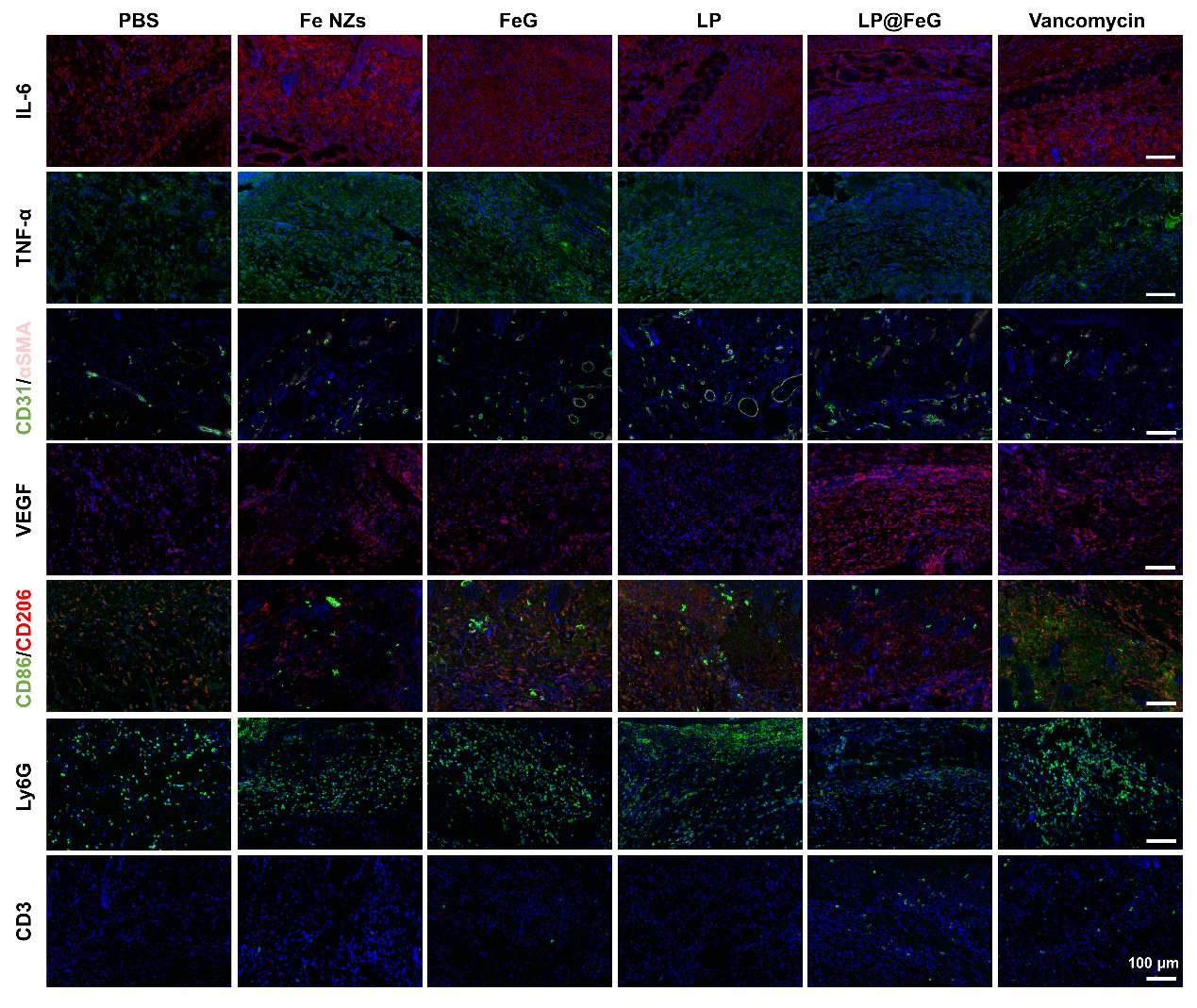


Fig.S30. Immunohistochemical staining for IL-6, TNF-α, CD31/αSMA, VEGF, CD86/CD206, Ly6G, and CD3 on Day 7.


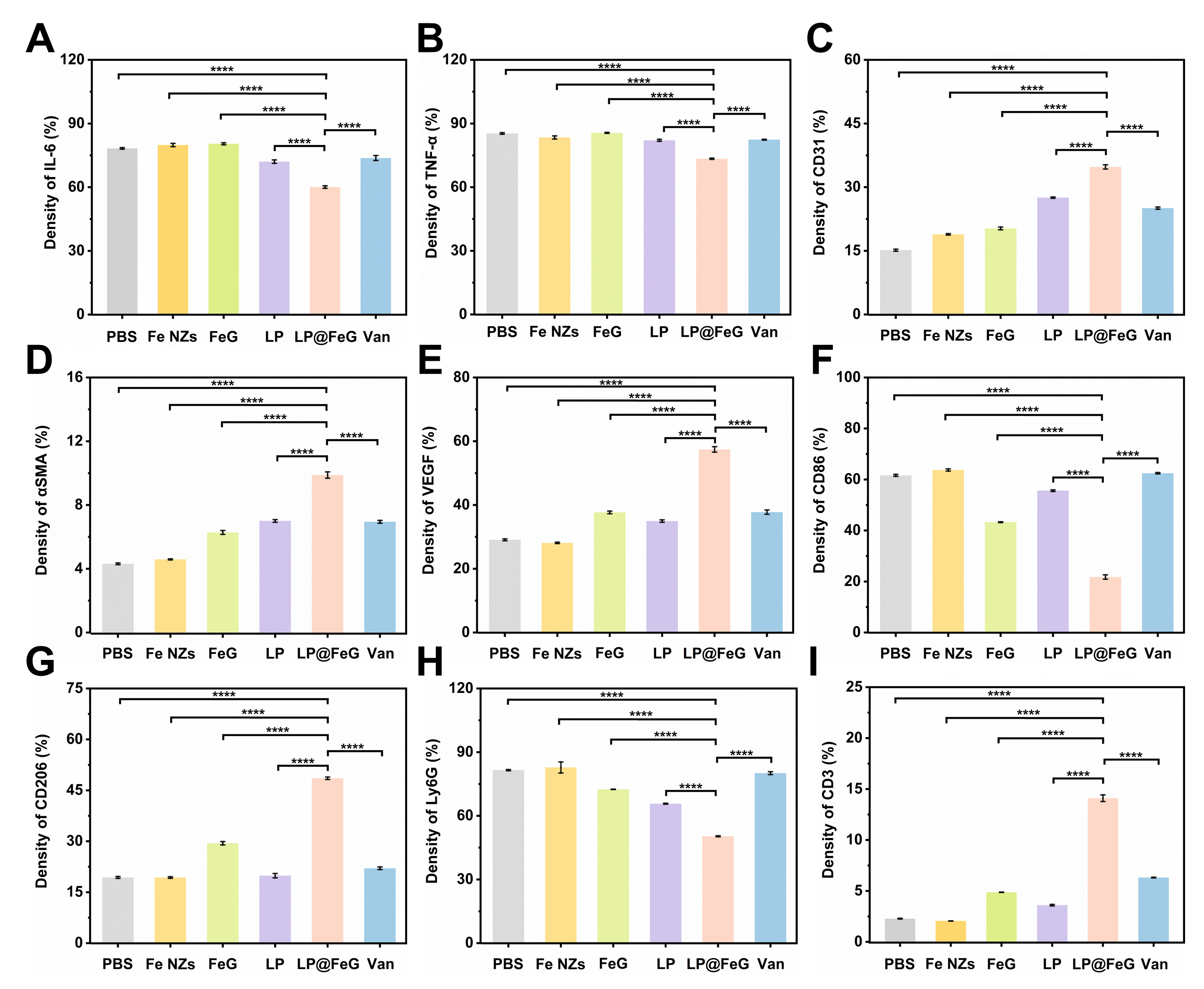


Fig.S31. Statistical analyses of (A) IL-6, (B) TNF-α, (C) CD31, (D) αSMA, (E) VEGF, (F) CD86, (G) CD206, (H) Ly6G, and (I) CD3 on Day 7.


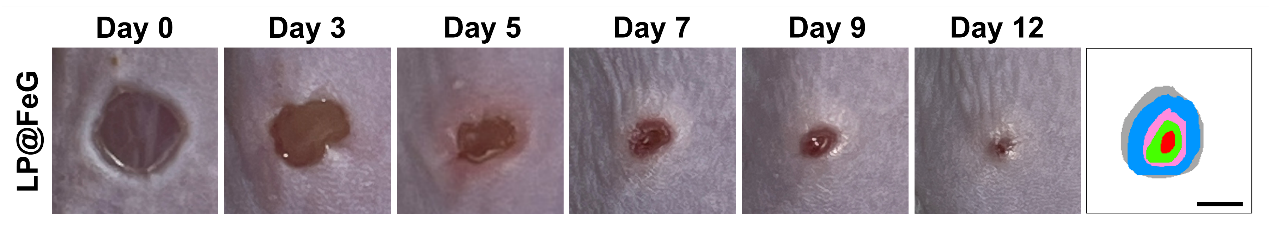


Fig.S32. Sequential photographs of wound healing in LP@FeG group after 0, 3, 5, 7, 9 and 12 days.


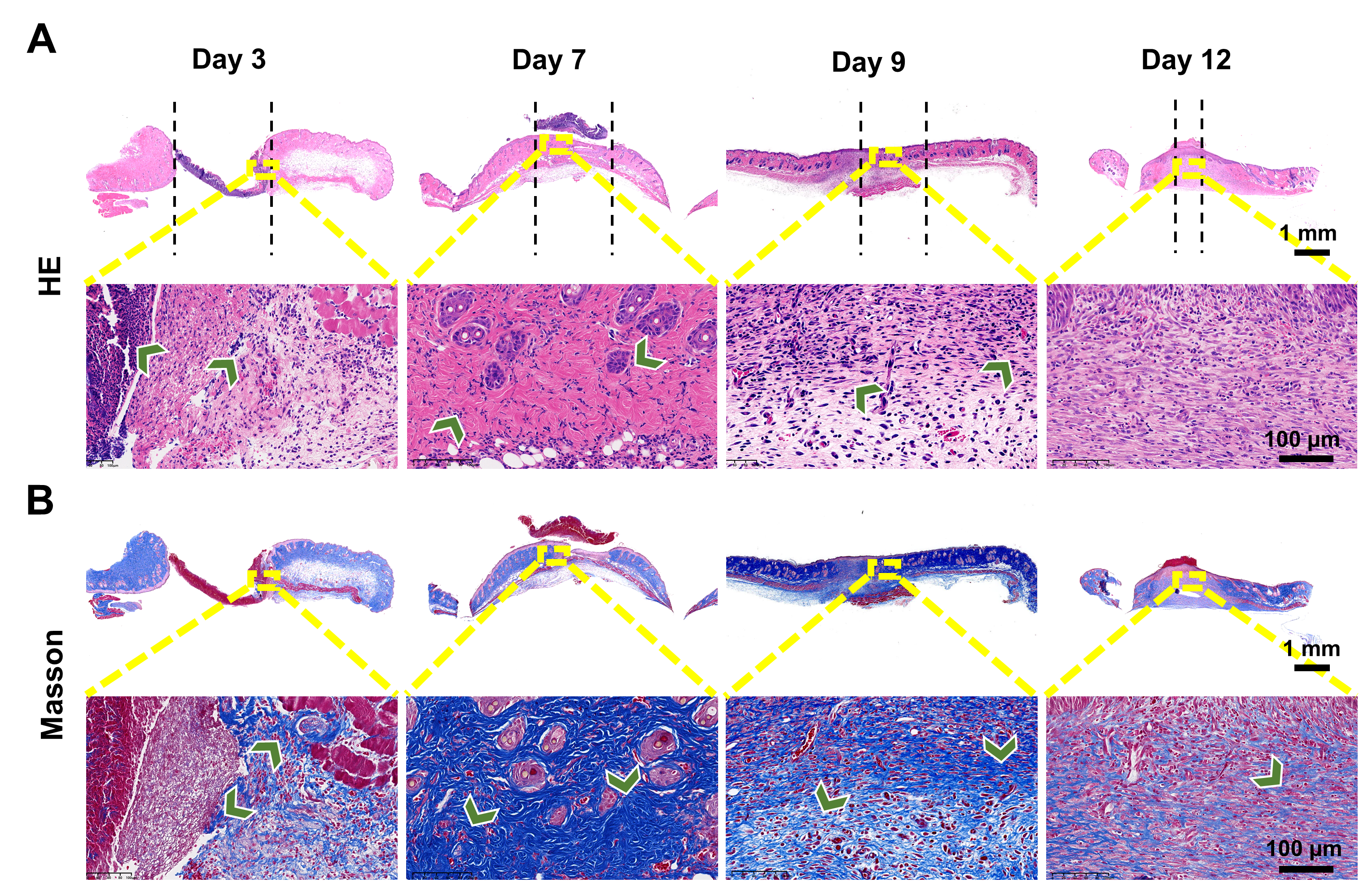


Fig.S33. (A) H&E images and (B) Masson staining images of wound tissues in LP@FeG groups after 0, 7, 9 and 12 days.


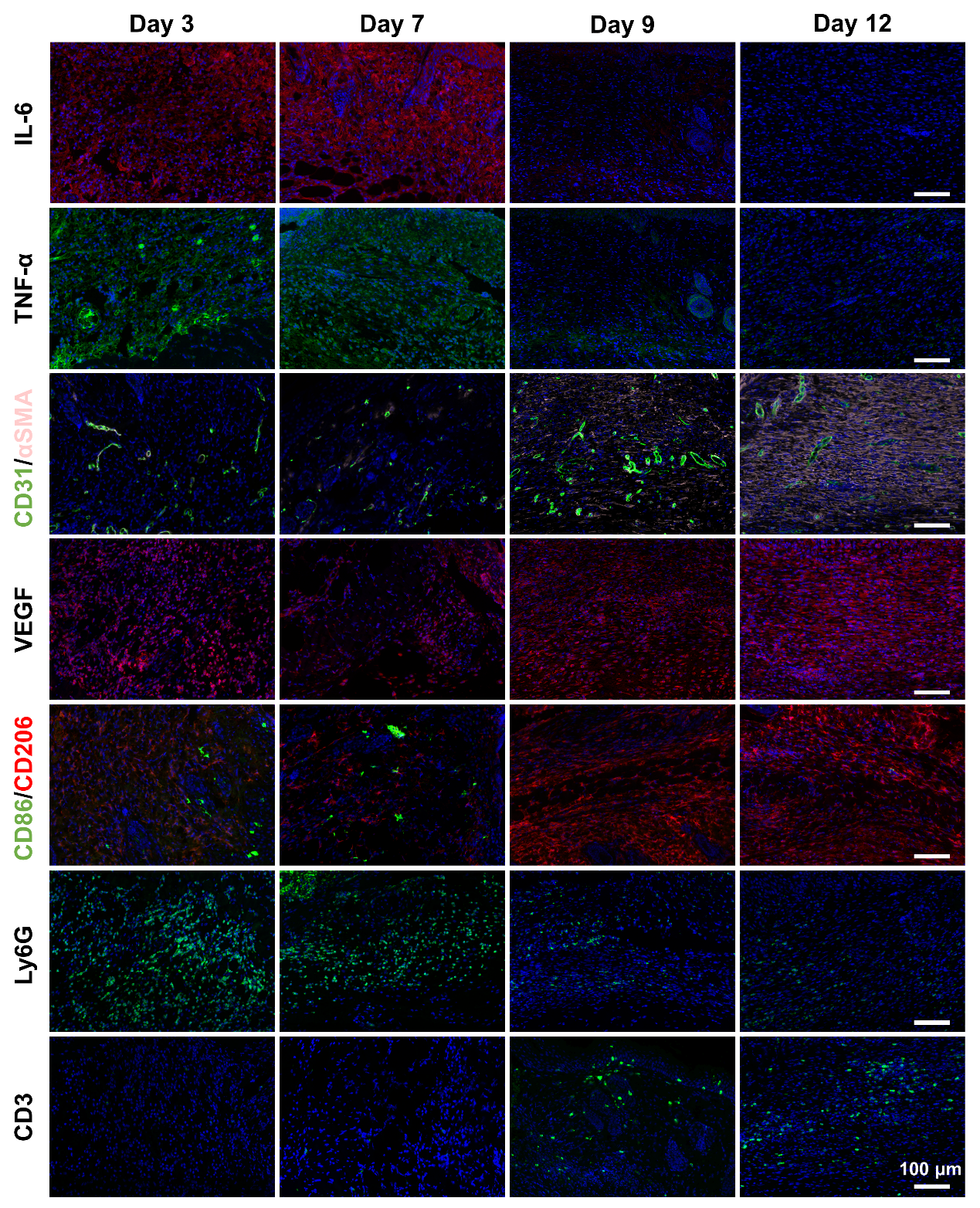


Fig.S34. Immunohistochemical staining for IL-6, TNF-α, CD31/αSMA, VEGF, CD86/CD206, Ly6G, and CD3 after 3, 7, 9 and 12 days.


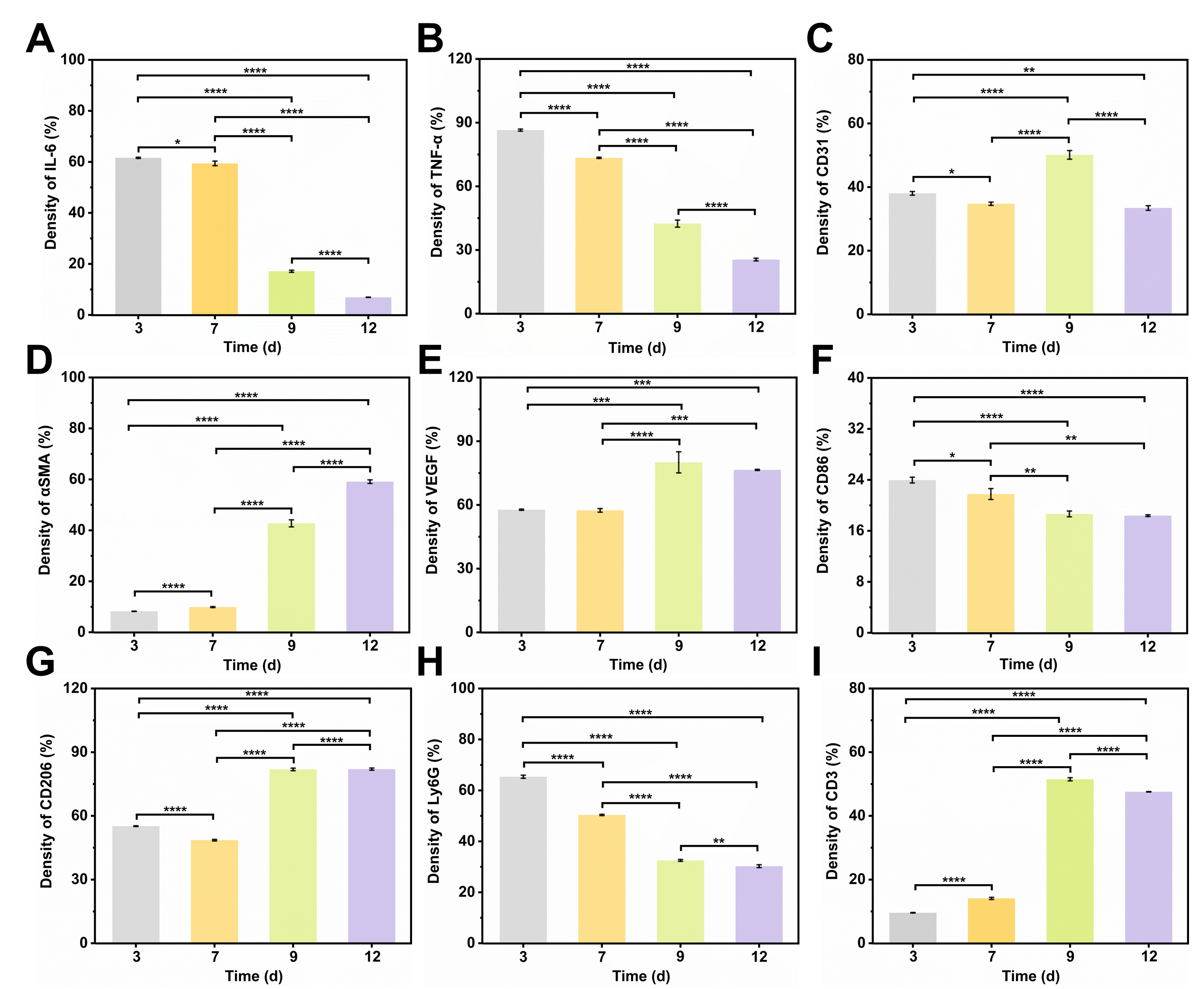


Fig.S35. Statistical analyses of (A) IL-6, (B) TNF-α, (C) CD31, (D) αSMA, (E) VEGF, (F) CD86, (G) CD206, (H) Ly6G, and (I) CD3 after 3, 7, 9 and 12 days.


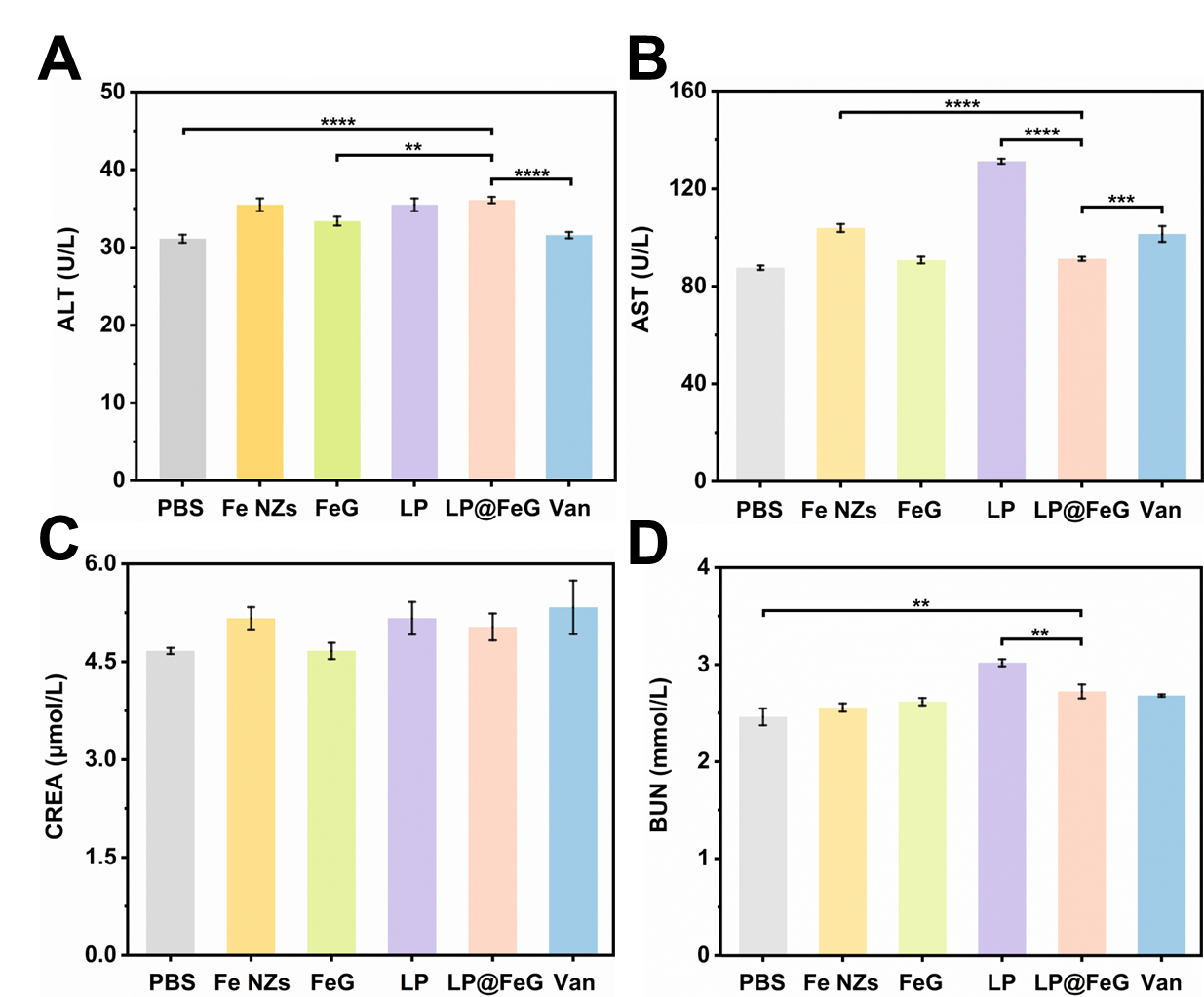


Fig.S36**.** Blood chemistry test including (A) ALT, (B) AST, (C) CREA, and (D) BUN of mice in different groups.


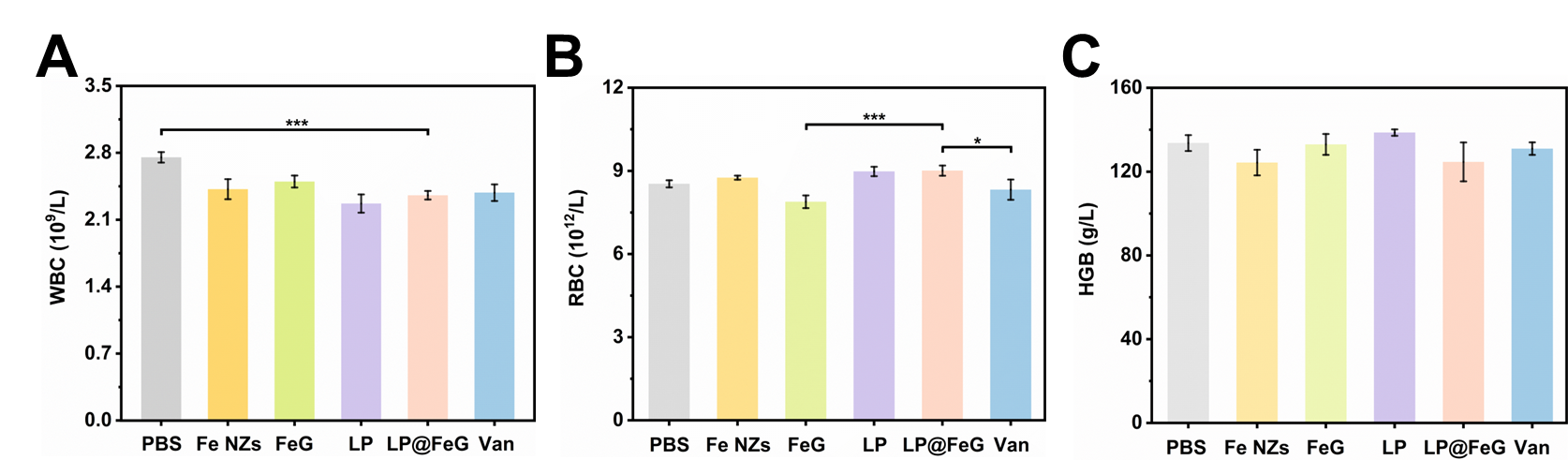


Fig.S37**.** Biochemical parameters including (A) WBC, (B) RBC, and (C) HGB of mice in different groups.


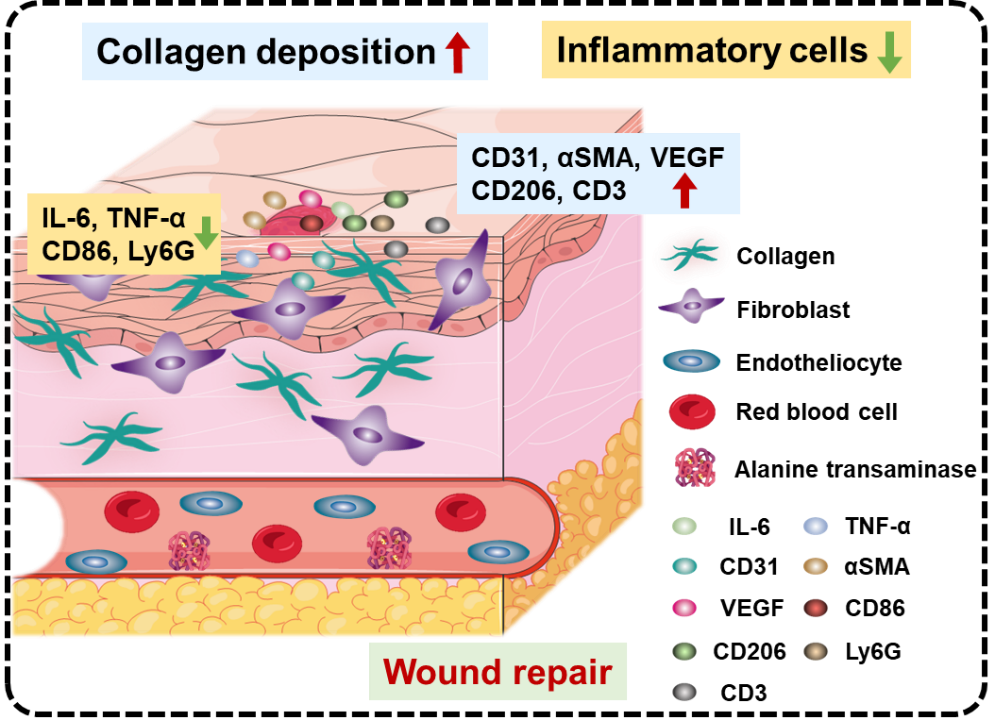


Fig.S38**.** Schematic illustration of the mechanism of LP@FeG in accelerating wound healing.

**
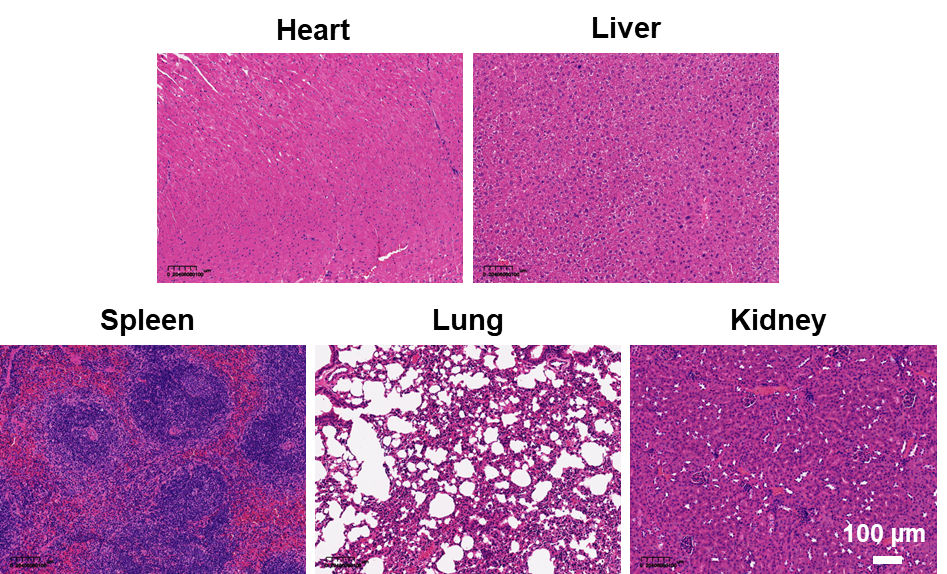
**

Fig.S39. H&E-stained sections of the major organs 30 days after treatment.

**
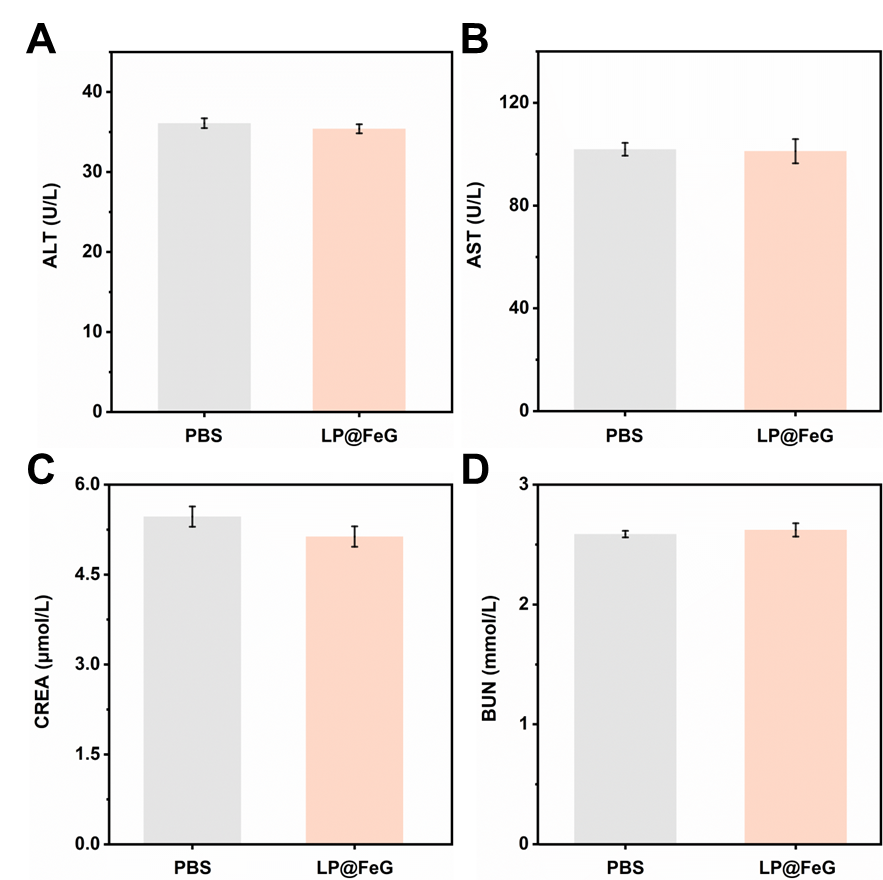
**

Fig.S40. Blood chemistry test including (A) ALT, (B) AST, (C) CREA, and (D) BUN of mice 30 days after treatment.

**
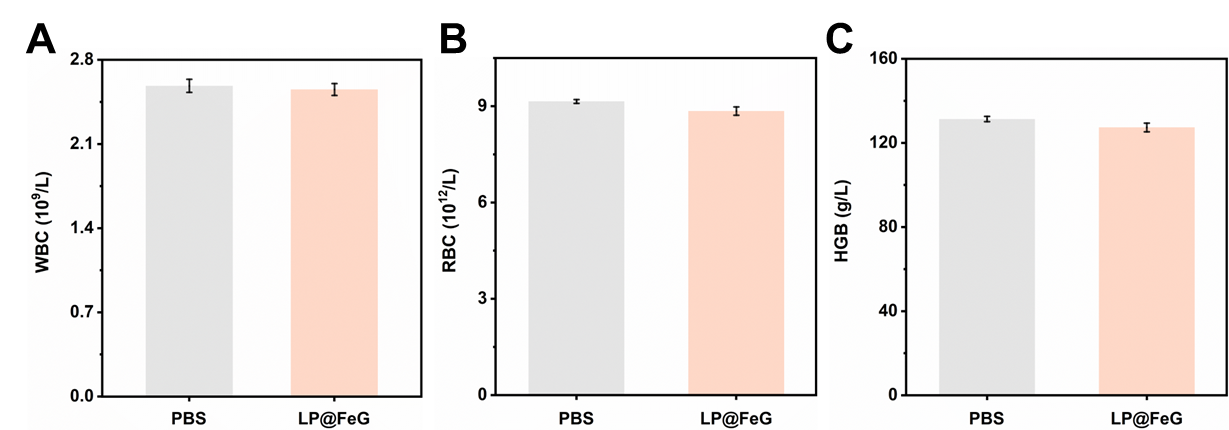
**

Fig.S41. Biochemical parameters including (A) WBC, (B) RBC, and (C) HGB of mice 30 days after treatment.

Table S1. Comparison of the *K_m_* and *V_max_* values for the POD-like activity of Fe NZs and other nanomaterials.

| Nanomaterials | *K_m_* (mM) | | *V_max_* (10^-7^ M/s) | |
| --- | --- | --- | --- | --- |
|  | H_2_O_2_ | TMB | H_2_O_2_ | TMB |
| **Fe NZs** | **2.944** | **1.426** | **12.797** | **10.412** |
| HRP [1] | 3.7 | 0.434 | 0.871 | 1 |
| Fe_3_O_4_ [1] | 154 | 0.098 | 0.978 | 0.344 |
| Ru@V_2_O_4_ [2] | 0.183 | 0.045 | 0.79 | 1.36 |
| PtFe@Fe_3_O_4_ [3] | 53.55 | 0.213 | 1.078 | 0.547 |
| MSN-AuNPs [4] | 15.81 | 0.0411 | 1.730 | 1.266 |
| Cu-HHTP [5] | 0.013 | - | 0.6257 | - |
| Cu-CeO_2_ [6] | 30.76 | - | 1.667 | - |
| PCNSs [7] | 471.00 | 2.25 | 0.552 | 0.447 |
| IP NPs-RSNO [8] | 0.204 | 0.025 | 5.62 | 8.48 |

**References**

[1] L. Gao, J. Zhuang, L. Nie, J. Zhang, Y. Zhang, N. Gu, T. Wang, J. Feng, D. Yang, S. Perrett, X. Yan. Intrinsic peroxidase-like activity of ferromagnetic nanoparticles. *Nat. Nanotechnol*. **2007**, 2, 577.

[2] J. Hou, P. Jia, K. Yang, T. Bu, X. Sun, L. Wang. Facile preparation of Ru@V_2_O_4_ nanowires exhibiting excellent tetra-enzyme mimetic activities for sensitive colorimetric H_2_O_2_ and cysteine sensing. *Sensor. Actuat. B: Chem*. **2021**, 344,

[3] S. Li, L. Shang, B. Xu, S. Wang, K. Gu, Q. Wu, Y. Sun, Q. Zhang, H. Yang, F. Zhang, L. Gu, T. Zhang, H. Liu. A Nanozyme with Photo-Enhanced Dual Enzyme-Like Activities for Deep Pancreatic Cancer Therapy. *Angew. Chem. Int. Edit*. **2019**, 58, 12624.

[4] Y. Tao, E. Ju, J. Ren, X. Qu. Bifunctionalized mesoporous silica-supported gold nanoparticles: intrinsic oxidase and peroxidase catalytic activities for antibacterial applications. *Adv. Mater*. **2015**, 27, 1097.

[5] M. Yang, Y. Liu, L. Zhang, Y. Qian, N. Li, G. Zhang, Y. Hu, X. Li, Y. Ge, Y. Peng, J. Ren, Y. Jiang, S. Guo, Y. Wang. Highly Conjugated Nanozyme with Non Coordination Saturation for Cascaded Enhanced POD Reaction Driving Antibacterial Therapy. *Adv. Funct. Mater.* **2024**,

[6] P. Jiang, L. Zhang, X. Liu, C. Ye, P. Zhu, T. Tan, D. Wang, Y. Wang. Tuning oxidant and antioxidant activities of ceria by anchoring copper single-site for antibacterial application. *Nat. Commun*. **2024**, 15, 1010.

[7] K. Fan, J. Xi, L. Fan, P. Wang, C. Zhu, Y. Tang, X. Xu, M. Liang, B. Jiang, X. Yan, L. Gao. In vivo guiding nitrogen-doped carbon nanozyme for tumor catalytic therapy. *Nat. Commun*. **2018**, 9, 1440.

[8] J. Hou, R. Fu, T. Yu, P. Ge, Y. Wang, M. Zhao, A. Zou, Y. Xianyu. Synergistic antibacterial therapy for multidrug-resistant bacterial infections using multifunctional nanozymes. *Nano Today* **2024**, 54.
